# The Influence of the Chemical Potential on Defects and Function of Perovskites in Catalysis

**DOI:** 10.3389/fchem.2021.746229

**Published:** 2021-09-17

**Authors:** Gregor Koch, Michael Hävecker, Pierre Kube, Andrey Tarasov, Robert Schlögl, Annette Trunschke

**Affiliations:** ^1^ Department of Inorganic Chemistry, Fritz-Haber-Institut der Max-Planck-Gesellschaft, Berlin, Germany; ^2^ Max Planck Institute for Chemical Energy Conversion, Heterogeneous Reactions, Max-Planck-Gesellschaft, Mühlheim, Germany

**Keywords:** SmMnO_3_, perovskite, surface modification, defects, heterogeneous catalysis, oxidative dehydrogenation of propane, AP-XPS, AP-NEXAFS

## Abstract

A Sm-deficient Sm_0.96_MnO_3_ perovskite was prepared on a gram scale to investigate the influence of the chemical potential of the gas phase on the defect concentration, the oxidation states of the metals and the nature of the oxygen species at the surface. The oxide was treated at 450°C in nitrogen, synthetic air, oxygen, water vapor or CO and investigated for its properties as a catalyst in the oxidative dehydrogenation of propane both before and after treatment. After treatment in water vapor, but especially after treatment with CO, increased selectivity to propene was observed, but only when water vapor was added to the reaction gas. As shown by XRD, SEM, EDX and XRF, the bulk structure of the oxide remained stable under all conditions. In contrast, the surface underwent strong changes. This was shown by AP-XPS and AP-NEXAFS measurements in the presence of the different gas atmospheres at elevated temperatures. The treatment with CO caused a partial reduction of the metals at the surface, leading to changes in the charge of the cations, which was compensated by an increased concentration of oxygen defects. Based on the present experiments, the influence of defects and concentration of electrophilic oxygen species at the catalyst surface on the selectivity in propane oxidation is discussed.

## Introduction

The A or B positions in the crystal lattice of ABO_3_ compounds with perovskite structure can be occupied by almost all metallic elements of the periodic table according to Goldschmidt’s rule (Goldschmidt, 1926). In this way, a wide variety of compounds is obtained. Other materials with special properties are accessible by partial substitution of either A or B leading to AA′BB′O_3_ formulas (Kurbakov, 2010; Zhu et al., 2015; Yang et al., 2016; Bartel et al., 2019). Chemical substitution is generally also accompanied by changes in defect structure. For example, the replacement of an A^3+^ with A^2+^ leads to lattice distortion (Kurbakov, 2010) and the formation of oxygen vacancies. Alternatively, partial oxidation of the B element may occur if this is possible (Ciambelli et al., 2000; McFarland and Metiu, 2013; Toniolo and Schmal, 2016). Both phenomena can also occur simultaneously. Defect formation can be achieved not only by the partial or complete substitution of A or B. Thus, reduction of the available amount of A or B also provides access to defect-rich compounds A_1-x_B_1-y_O_3_ that differ significantly from the ideal stoichiometry ABO_3_ (Golikov et al., 2005; Neagu et al., 2013). Due to the dense packing of ions in the perovskite structure, the occupation of interstitial sites is very unlikely. Therefore, for defect formation, only vacancy formation or migration of smaller B elements to vacant A positions, which offer more space, can be considered (Wołcyrz et al., 2003; Ignatans et al., 2019).

Defect-rich structures are of particular importance with respect to the application of perovskites in heterogeneous catalysis (Royer et al., 2014; Najjar and Batis, 2016). In addition to the increasing interest in perovskites as catalysts for oxygen reduction and oxygen evolution reactions (Suntivich et al., 2011; Gupta et al., 2016; Zhu et al., 2019), this class of materials is also widely used in photocatalysis (Grabowska, 2016; Tasleem and Tahir, 2020) or total oxidation to remove volatile organic and hazardous compounds (Gil et al., 2004; Liu et al., 2018a; Liu et al., 2018b; Liu et al., 2019). However, perovskites are also suitable catalysts for selective oxidations at low temperatures (Polo-Garzon and Wu, 2018; Kamata, 2019), especially for the oxidative dehydrogenation of propane (Koch et al., 2020). The catalytic properties can be influenced by various factors, such as the surface composition (Fierro and Tejuca, 1987; Koch et al., 2020), the concentration of defects, or the partial reduction of metal ions (Evarestov et al., 2005; Mierwaldt et al., 2014; Ignatans et al., 2019; Koch et al., 2020). Oxygen species at the catalyst surface are responsible for the activation of the propane molecule. X-ray Photoelectron Spectroscopy (XPS) allows the identification of different oxygen species in the surface region of perovskites (Mierwaldt et al., 2014; Stoerzinger et al., 2014; Stoerzinger et al., 2015). Furthermore, the dynamics of these species can be studied as a function of the chemical potential of the gas phase under reaction conditions of the selective oxidation of propane using ambient pressure (AP) XPS (Koch et al., 2020).

Mn-based perovskites are generally catalyzing the total combustion of propane already at low reaction temperatures. However, the addition of water vapor to the feed of propane and oxygen leads to higher selectivity of the desired selective oxidation product propene. The improved selectivity is apparently related to the partial reduction of surface manganese ions and the formation of defects and hydroxides (Koch et al., 2020). To examine these relationships in more detail, a samarium-deficient Sm_0.96_MnO_3_ perovskite was prepared by combustion synthesis and subjected to various post-treatments at 450°C in inert gas and gases containing H_2_O and CO. The compound was chosen because it is structurally stable under relevant reaction conditions (Kamata et al., 1979; Hui et al., 1990; Golikov et al., 2005) and exhibits good catalytic activity at low temperatures as well as significant changes in catalytic performance upon variation of the feed composition. In contrast, LaMnO_3_ (Koch et al., 2020), which is often studied in oxidation reactions, is more sensitive to changes in oxygen partial pressure. This can ultimately lead to defect restructuring associated with the transformation of crystal symmetry from rhombohedral to orthorhombic and thus with the change of bulk structure (Miyoshi et al., 2003).

The catalyst was examined before and after the treatments in the different gas phases with respect to its catalytic performance, defect structure and surface composition. AP-XPS experiments and ambient pressure Near Edge X-Ray Absorption Fine Structure (AP-NEXAFS) measurements in the presence of the gas phase were performed to study in detail the changes in the O 1s spectra and the surface oxidation state of the Mn and Sm ions as a function of the pretreatment conditions and the feed composition. Based on the spectroscopic results, possible relationships between the surface structure of the perovskite and its properties as a catalyst in propane oxidation are discussed.

## Materials and Methods

### Synthesis

A self-igniting gel was first prepared by the Pecchini method (Pechini, 1967). Glycine was used as a fuel in a stoichiometric ratio to the metal nitrate concentration, so that combustion occurred after ignition of the resulting gel without the participation of oxygen from the gas phase (Cooper and Kurowski, 1996; Hwang et al., 2005; Koch et al., 2020). At first, 13.74 g of Sm(NO_3_)_3_·6H_2_O (Aldrich, 99.9% trace metal basis, LOT: MKCG 1993) were dissolved in 75 ml H_2_O. Afterward, 8.17 g of Mn(NO_3_)_2_·4H_2_O (ROTH, 98%, LOT: 46827460) were added, yielding a slightly yellow solution. While stirring 6.27 g of glycine (Lancester Synthesis, Batch 10052309) was added. After stirring for 1 h, the solution was transferred into an evaporation basin placed on a hot plate and the water was evaporated at 120°C. The formed resin was self-ignited by heating to 350°C. The dark powder (ID 29987) was obtained with a yield of 82%. On basis of thermogravimetry, the optimal calcination temperature was determined (Supplementary Figure S1). For calcination, 6.27 g of combustion product was put into a combustion boat which was placed in a tube furnace. While streaming in 200 ml min^−1^ a mixture consisting of 20% O_2_ in Ar, the powder was heated to 800°C at 5°C min^−1^, calcined at this temperature for 5 h, and then cooled to 25°C. The calcination yield was 91%, which corresponded to an overall yield of 76%. The catalyst ID is 30036.

### X-Ray Diffraction

X-ray diffraction (XRD) patterns were measured on a Bruker D8 ADVANCE II theta/theta diffractometer, using Ni-filtered Cu Kα radiation and a position-sensitive LynxEye silicon strip detector. Structure parameters were calculated by a least-square fitting of the diffraction patterns by using corresponding structure models taken from the ICSD database. Refinement was performed utilizing the program package TOPAS (version 4.2, copy-right 1999–2009 Bruker AXS).

### X-Ray Fluorescence

The metal content of the perovskite catalysts was determined by X-ray fluorescence spectroscopy using a Bruker S4 Pioneer wavelength dispersive X-ray fluorescence spectrometer. For sample preparation, a mixture of 0.05 g of the catalyst and 8.9 g of lithium tetraborate (>99.995%, Aldrich) was fused into a disk using an automated fusion machine (Vulcan 2 MA, Fluxana).

### Thermogravimetry Coupled with Mass Spectrometry

The weight loss of the powder was measured using a NETZSCH TG 209F1 Libra thermo balance and the gas phase composition was determined using a Pfeiffer Omnistar mass spectrometer. 11.782 mg of the sample were put into an Al_2_O_3_ crucible, which was then placed on the sample holder. After equilibration time, the sample was heated to 900°C at 10°C min^−1^ while a gas flow of 70 ml min^−1^ streamed through the heating chamber. The gas was composed of 21% O_2_ in Ar. Previously measured correction without sample was considered in data analysis.

The same setup was used for the TG-MS experiment simulating the CO treatment. 11.873 mg of the calcined sample were loaded in an Al_2_O_3_ crucible. After equilibrating, the calcined sample was heated to 450°C at 10°C min^−1^ followed by a dwelling period of 20 min. The gas consisted of 21% O_2_ in Ar and streamed with 70 ml min^−1^ to remove all surface carbonates possibly formed. Subsequently, the atmosphere was changed to 2% CO in Ar at the same flow rate and the temperature program was repeated.

### N_2_ Physisorption

N_2_ adsorption isotherms were measured at −196°C using the Autosorb-6B analyzer (Quantachrome) after outgassing the catalysts in the vacuum for 2 h at 150°C. Data analysis was done using the Quantachrome Autosorb software package. The specific surface area was calculated according to the multipoint Brunauer-Emmett-Teller method (BET) in the range 0.05 < *p*/*p*_0_ < 0.15 assuming the N_2_ cross-sectional area of 16.2 Å^2^.

### Scanning Electron Microscopy and Energy Dispersive X-Ray Fluorescence Spectroscopy

The sample was fixed on carbon tape on the top of an Al-sample stage before the transfer into the SEM load chamber. SEM images were conducted using a Hitachi S-4800 microscope equipped with a cold field emission gun. For imaging, 1.5 kV acceleration voltage and 4 mm working distance was set to display the image by using both, upper and lower secondary electron detector. Additionally, EDX mapping was done setting the acceleration voltage to 15 kV and working distance to 10 mm. An energy-dispersive QUANTAX 800 EDX spectrometer working with a XFlash^®^6│30 detector was used to map the elemental distribution. Usually, mapping was done for 2 min when sufficient counts were gained (>10 kps). Recorded spectra were analyzed considering all found peaks and corresponding elemental ratios were calculated for each map/spectrum. Standard deviations were calculated on the results of all maps.

### Catalytic Testing

The catalytic tests were carried out as described before (Koch et al., 2020) using a setup for partial oxidation (Integrated Lab Solutions) with 10 fixed bed tubular reactors in parallel. All reactors have an inner diameter of 2 mm and all reactors were equipped with a thermocouple placed inside the catalyst bed. The catalytic performance was determined at atmospheric pressure under steady-state conditions. The reactant feed consisted of 5% propane and 10% O_2_ in N_2_ (C_3_H_8_/O_2_/N_2_ = 5/10/85). Furthermore, 40% H_2_O was added to the feed using an evaporator (C_3_H_8_/O_2_/N_2_/H_2_O = 5/10/45/40). Before testing, the catalysts were pressed with a force of 2 t for 1 min, crushed, and sieved to particles, which sizes ranged from 100 to 200 µm. Generally, a total flow of 15 ml min^−1^ was set. Mass of catalysts of 52, 96 and 142 mg were loaded into single steel reactors to achieve contact times of W/F = 0.22 g∙s∙ml^−1^, W/F = 0.38 g∙s∙ml^−1^ and W/F = 0.57 g∙s∙ml^−1^.

An online gas chromatograph (Agilent 7890A) was used for gas analysis. A combination of Plot-Q (length 30 m, 0.53 mm internal diameter, 40 µm film thickness) and Plot-MoleSieve 5 A columns (30 m length, 0.53 mm internal diameter, 50 µm film thickness), connected to a thermal conductivity detector (TCD), was used to analyze the permanent gases CO, CO_2_, N_2_, O_2_, and CH_4_. A system of an FFAP (length 30 m, 0.53 mm internal diameter, 1 µm film thickness) and a Plot-Q column (length 30 m, 0.53 mm internal diameter, 40 µm film thickness) connected to a flame ionization detector (FID) was used to analyze the C2-C3 hydrocarbons.

Conversion of propane $X_{C_{3}H_{8}}$, and selectivity *S*_*i*_ of product *i* in percentage, were calculated based on the carbon number and the sum of all products formed:

$$X_{C_{3}H_{8}}=\frac{\sum_{i=1}^{n}N_{i}c_{i}}{\sum_{i=1}^{n}N_{i}c_{i}+3c_{C_{3}H_{8},out}}\cdot 100$$

$$S_{i}=\frac{N_{i}c_{i}}{\sum_{i=1}^{n}N_{i}c_{i}}\cdot 100,$$

where *N*_*i*_ is the number of carbon atoms in the product *i*, *c*_*i*_ is the concentration of the product *i* in the reactor exit gas, and $\;c_{C_{3}H_{8},out}\;$ is the concentration of propane in the exit gas. The conversion of oxygen in percentage was calculated based on the difference between the oxygen concentration in the reactant feed $c_{O_{2},in}$ and in the exit gas $c_{O_{2},out}$:

$$X_{O_{2}}=\frac{c_{O_{2},in}-c_{O_{2},out}}{c_{O_{2},in}}\cdot 100.$$

Integral reaction rates *r*_propane_ normalized to the specific surface area in µmol∙h^−1^∙m^−2^ for propane consumption were determined based on the first derivation of the linear function

$$X_{C_{3}H_{8}}=f\left(\frac{W}{F}\right),$$

resulting in:

$$r_{propane}=\frac{dX_{C_{3}H_{8}}}{d\left(\frac{W}{F}\right)S},$$

where $X_{C_{3}H_{8}}$ is used not in percentage, but as a value between 0 and 1, *W* is the catalyst mass in g, *F* is the flow rate of propane in µmol∙h^−1^, and *S* is the specific surface area in m^2^∙g^−1^.

Initial formation rates *r*_initial_ of propene and CO_2_ were calculated through extrapolation. Corresponding formation rates at each contact time were fitted with a linear function. The function was used to calculate the initial rates at 0% conversion of propane.

### Ambient Pressure Photoelectron Spectroscopy and Ambient Pressure Near-Edge X-Ray Absorption Spectroscopy

Spectra were collected according to the procedure described in (Koch et al., 2020). AP-XPS and AP-NEXAFS measurements were performed at the ISISS (Innovative Station for *In Situ* Spectroscopy) facility operated by the MPG at the synchrotron radiation source BESSY II in Berlin, Germany. 12.2 mg of sample was pressed into a pellet of 8 mm in diameter. The pellet was mounted on a steel plate and covered by a steel cover lid having a 4 mm central aperture. The sample was connected to a thermocouple and then the sample holder was loaded to the instrument as described previously (Salmeron and Schlögl, 2008; Knop-Gericke et al., 2009).

Firstly, spectra were collected at room temperature in a vacuum, then heated in a stream of 5 ml⋅min^−1^ atmosphere consisting of propane/O_2_/He/H_2_O of 0/20/80/0 (heating rate 5°C min^−1^) to 270°C. All gases and water vapor were dosed to the XPS cell via calibrated mass flow controllers (BRONKHORST). The gas composition was then switched consecutively to 0/10/90/0, and 5/10/45/40 before switching to 25% CO in He. This was followed by heating to 450°C at 5°C min^−1^. After spectra collection, the sample was cooled to 270°C at 5°C min^−1^ again and the atmosphere was set to 5/10/45/40, and then to 5/10/85/0. The total gas pressure at all reaction conditions was 0.25 mbar.

AP-XPS spectra were collected with an exit slit width of the beamline of 111 μm. The pass energy of the hemispherical electron analyzer was 20 eV for all core level spectra. The photon energy scale of the beamline has been adjusted by measuring the Fermi edge of a Pd foil at various photon energies. The measurements were carried out at a constant photoelectron kinetic energy of ≈170 eV for all core levels, examining the outermost surface layer with an approximate thickness corresponding to an approximate electron inelastic mean free path IMFP of *λ* = 0.6 nm. For this purpose, the photon energies were set to 255 eV (Mn 2s), 810 eV (Mn 2p), 700 eV (O 1s), 455 eV (C 1s), 1,260 eV (Sm 3d), 315 eV (Sm 4d) and 170 eV (valence band). Hence, the transmission of electrons through the gas phase and in the lens of the electron spectrometer was the same for all elements and does not need to be included in the quantitative calculation of element abundances. Additionally, the subsurface region of the material was studied by using the larger kinetic energy of ≈770 eV using photon energies of 1,300 eV (O 1s) and 1,055 eV (C 1s) that corresponds to an IMFP of λ = 1.6 nm. These values of *λ* were estimated using the predictive TPP-2M formula by Tanuma, Powell and Penn (Tanuma et al., 1991; Tanuma et al., 2003) (Powell and Jablonski, 2010) (NIST Electron Inelastic-Mean-Free-Path Database Version 1.2). For the quantitative analysis of the core level intensities, the atomic subshell photoionization cross sections and asymmetry parameters from numerical calculations by Yeh and Lindau (Yeh and Lindau, 1985; Yeh, 1993) were used taking the monochromatic photon energy-dependent photon flux into account. The core-level spectra were deconvoluted using Gaussian-Lorentzian product functions after subtracting a Shirley background with the CasaXPS software (Neal Fairley, Version 2.3.15, ^©^ 1999–2009 Casa Software Ltd.). Gas-phase peaks have been suppressed during the AP-XPS measurements by applying a bias of +90 V to the entrance aperture of the spectrometer.

AP-NEXAFS data processing included averaging two spectra of each edge followed by background subtraction and normalization. Total electron yield was used for Mn L-edge spectra and Sm M-edge spectra. Auger electron yield was used for O K-edge spectra with the analyzer set to a kinetic energy of *E*_*kin*_ = 385 eV and a pass energy of *E*_*pass*_ = 50 eV.

## Results

Phase-pure Sm_0.96_MnO_3_ (Supplementary Figure S2, Table 1) ICSD# 95491 was prepared by the self-combustion synthesis route (Mori et al., 2002). The catalyst exhibited pores and voids of different sizes due to the high amounts of gases formed during ignition synthesis. The pores were retained during calcination. Thermal treatment of the synthesized material in oxygen is generally required to remove remaining organic components and carbonates formed by storage in air (Supplementary Figure S1). During the calcination of the combustion product, small crystallites were formed that adhere to each other and creating cavity-rich macrostructures (Supplementary Figure S3). The predominant size fraction of the nanocrystals was about 50 nm according to SEM, which is in good agreement with the calculated crystallite size from XRD refinement (51 nm, Table 1). However, larger and smaller crystallites are also present, but only in small fractions (Supplementary Figure S3). Due to the irregular macrostructure and the voids, a specific surface area of 7.6 m^2^⋅g^−1^ was obtained (Table 2). The desired non-stoichiometry of Sm and Mn in the volume was reached by using a reduced mass of the samarium precursor (Table 2). However, the surface of the catalyst showed a 1:1 stoichiometry of Sm and Mn (Table 2). Analysis of the volume revealed a slight oxygen deficit of the compound (Sm_0.96_MnO_2.93_, Table 2). Therefore, the segregation of nano-sized binary oxides seems unlikely (Golikov et al., 2005; Neagu et al., 2013). Segregated phases were also not detected by XRD (Supplementary Figure S2) and EDX mapping (Supplementary Figure S4).

**TABLE 1:** Lattice parameters obtained form XRD analysis of fresh and spent Sm_0.96_MnO_3_ catalysts.

	Fresh	Spent
Space group	*Pnma*	*Pnma*
*a*/Å	5.82773 (15)	5.83812 (12)
*b*/Å	7.4979 (2)	7.49057 (16)
*c*/Å	5.36351 (15)	5.363,300 (12)
*V*/Å^3^	234.362 (11)	234.529 (9)
Occupancy Mn	1.108 (9)	1.126 (9)
Occupancy Sm	1.069 (9)	1.086 (9)
Size (LVol-IB)/nm	51.2 (12)	47.1 (9)

**TABLE 2:** Surface area and elemental composition of Sm_0.96_MnO_3_ obtained from bulk analysis (XRF and O-content) and surface analysis (XPS and NEXAFS).

Parameter	Value or formula
Surface area / m^2^⋅g^−1^	7.6
Mn/Sm (XRF)	1.05
Oxygen content/wt%	19.09 ± 0.57
Chemical composition (normalized to O_3_)	Sm_0.98_Mn_1.02_O_3.00±0.09_
Formula (normalized to Sm)	SmMn_1.05_O_3.06±0.09_
Formula (normalized to Mn)	Sm_0.96_MnO_2.93±0.09_
Mn oxidation state (based on O-content (XRF))	2.99 ± 0.18
Mn/Sm (EDX)	1.11 ± 0.01
Mn/Sm (Lab-XPS)	1.02
Mn/Sm (AP-XPS)	1.01
Mn oxidation state (XPS Mn 3s splitting) Galakhov et al. (2002)	3.09
Mn oxidation state (NEXAFS), Koch et al. (2020)	3.03

**FIGURE 1:**
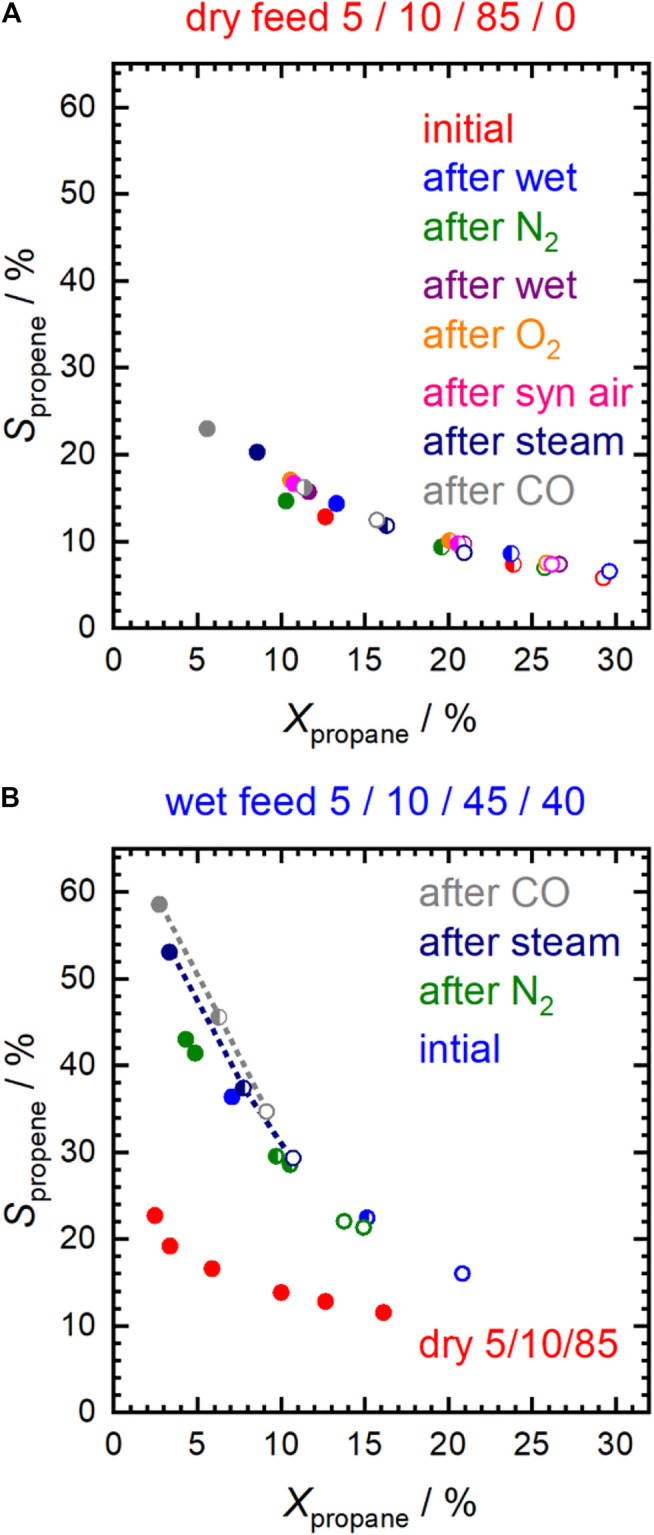
Selectivity to propene in **(A)** dry feed (C_3_H_8_/O_2_/inert = 5/10/85) and **(B)** in wet feed (C_3_H_8_/O_2_/inert/H_2_O = 5/10/45/40) after specific pretreatments at 450°C. Additionally, in **(B)** selectivity at different conversions in dry feed in the temperature range from 250 to 310°C is depicted for comparison (red data points). F/W = 0.29 L⋅min^−1^∙g^−1^ (full symbols); F/W = 0.16 L⋅min^−1^∙g^−1^ (half-filled symbols); F/W = 0.11 L⋅min^−1^∙g^−1^ (empty symbols).

The Sm_0.96_MnO_3_ catalyst is oxidizing propane already at temperatures below 300°C. (Merino et al., 2005; Koch et al., 2020). In the present work, the reaction was performed in either dry feed, i.e. 5% propane and 10% O_2_ in nitrogen (5/10/85), or wet feed, i.e. 5% propane, 10% O_2_ and 40% H_2_O in nitrogen (5/10/45/40) (Koch et al., 2020). The dry feed at 300°C was chosen as a reference point to compare the influence of the different pretreatments on activity and selectivity (Supplementary Figure S5). The selectivity to propene measured at the reference points as a function of propane conversion is shown in Figure 1A for different contact times. Regardless of the different pretreatment conditions, represented by different colors, and regardless of the contact time, represented by full, half-filled and empty symbols, all the measurement points lie on the same S-X curve. Thus, in dry feed, it can be seen that the different pretreatments only affected the integral rates. Compared to the conversion after calcination in air at 450°C, the conversion decreased slightly after treatment in pure N_2_ at the same temperature. This loss of activity could not be reversed by subsequent treatment in pure oxygen and further treatment in synthetic air at 450°C. The decreased conversion could be caused by either slight sintering or surface modifications. An increase in the apparent activation energy from 61 ± 6 kJ⋅mol^−1^ to 76 ± 4 kJ⋅mol^−1^ after N_2_ pretreatment suggests more for the latter (Supplementary Table S1). After the treatments in 10% water vapor and 2% CO, the activity decreases more significantly.

**FIGURE 2:**
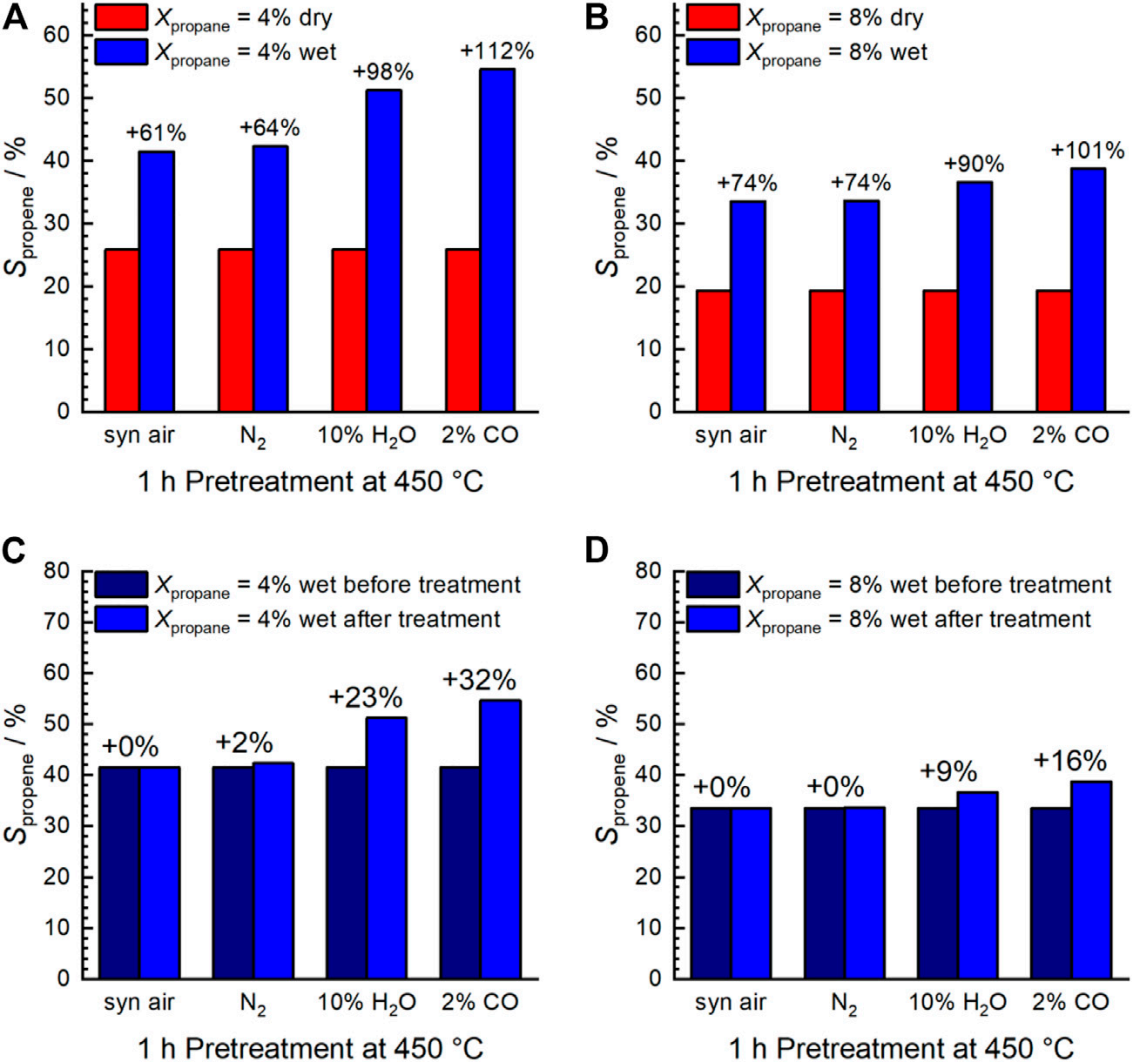
Selectivity to propene shown at the same conversion of propane: 4% **(A,C)** and 8% **(B,D)**. Comparison between dry and wet feed **(A,B)** and initial wet feed (after calcination in air at 450°C) and wet feed after the different treatments **(C,D)**. C_3_H_8_/O_2_/inert = 5/10/85 (dry feed); C_3_H_8_/O_2_/inert/H_2_O = 5/10/45/40 (wet feed); F/W = 0.29 L min^−1^∙g^−1^; T = 300°C. The numbers above the columns indicate the respective increase in selectivity.

Using wet feed, the selectivity to propene remarkably increased significantly (by about 18–83% over the entire range of measured conversions) at comparable conversion (Figure 1B) indicating a change in the properties of the catalyst probably related to the addition of water vapor. An increase in selectivity in the wet feed has been observed previously for Mn-based perovskites that had a nearly balanced Mn/A element ratio or an excess of Mn at the surface (Koch et al., 2020). In contrast, an Mn deficiency on the surface resulted in reduced selectivity (Koch et al., 2020). This effect was particularly pronounced after the pretreatments in steam and CO at 450°C, and the selectivity improvement was more evident at low propane conversion (Figures 2A,B). Apparently, both steam treatment and reducing conditions during pretreatment promote the formation of a more selective catalyst under reaction conditions, but only when propane oxidation is carried out in the presence of H_2_O in the feed. When switched to dry feed, the catalyst returns to its non-selective mode (Figure 1A and Figures 2A,B).

The used catalyst showed the same bulk structural properties as the fresh catalyst (Table 1, Supplementary Figure S2). Only minor changes in lattice parameters were observed, which did not affect the total unit cell volume. Therefore, an extensively increased formation of oxygen vacancies in the bulk could be excluded, because this should have led to lattice expansion and increased cell volume (Miyoshi et al., 2003). Moreover, the excess of Mn in the bulk remained after catalytic testing (data not shown). Recrystallization or crystallite growth did not occur at these low temperatures applied because the crystallite sizes before and after catalytic testing were similar.

In contrast to the stable bulk structure, the surface structure changed significantly when the chemical potential was varied, which was achieved both by changing the feed and varying the pretreatment atmosphere. To understand the significant increase in selectivity to propene in the wet feed after pretreatment with carbon monoxide (Figure 1B and Figures 2C,D), the surface was analyzed under reaction conditions.

**FIGURE 3:**
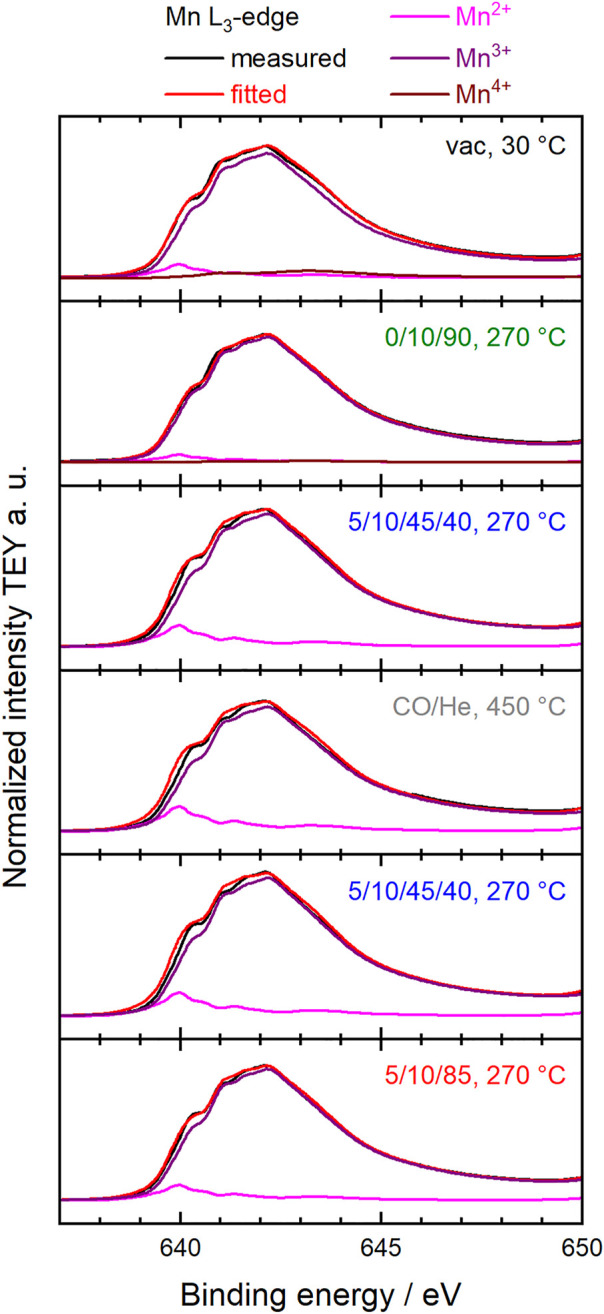
Measured and normalized TEY Mn L_3_-edges (black) and corresponding fitted spectra (red) using a linear combination of reference spectra of Mn^2+^ (pink), Mn^3+^ (purple) and Mn^4+^ (brown) (Koch et al., 2020). The conditions during the measurements at the synchrotron facility are indicated in each diagram in terms of temperature and feed compositions C_3_H_8_/O_2_/He or C_3_H_8_/O_2_/He/H_2_O.

**FIGURE 4:**
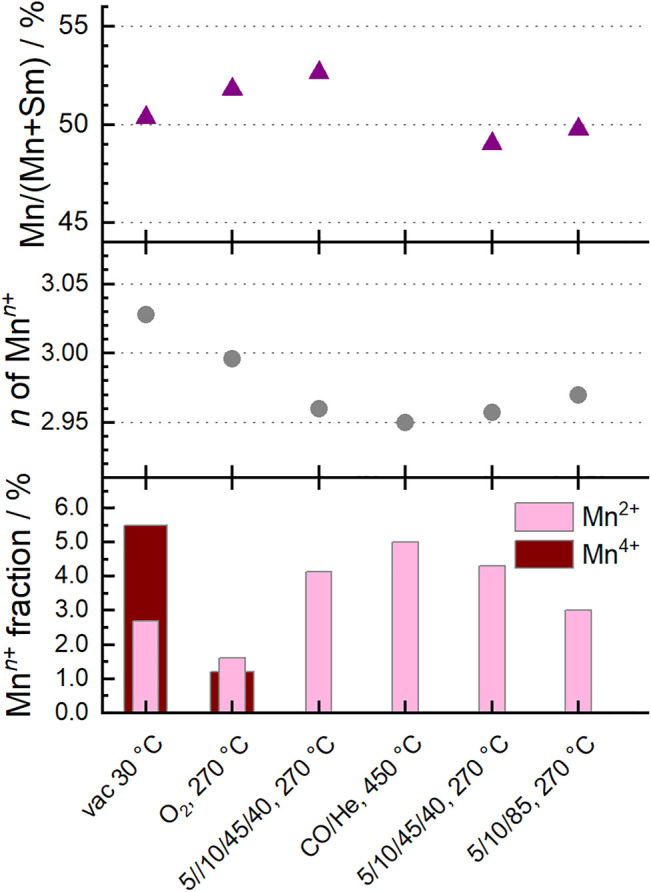
Surface characteristics of Sm_0.96_MnO_3_ at 0.25 mbar at different temperatures and different feeds (C_3_H_8_/O_2_/He/H_2_O) as given on the *x*-axis. Bottom: Fraction of Mn^2+^ and Mn^4+^ of all Mn ions measured with NEXAFS. Values obtained from NEXAFS Mn L_2,3_-edge fitting. Mn^3+^ balances the rest. Middle: Corresponding average oxidation state *n* of Mn^*n*+^. Top: Mn fraction obtained from Mn 2p and Sm 3d XPS peak area analysis.

Fitting the Mn L_3,2_-edges using a linear combination of relevant reference spectra for Mn^2+^, Mn^3+^ and Mn^4+^ species in the perovskite structure (Koch et al., 2020) showed that the oxidation state of manganese in the surface region changed under the different conditions (Figure 3). The dominant oxidation state of manganese in perovskites is Mn^3+^, accompanied by smaller proportions of Mn^2+^ and Mn^4+^ (Figure 4). (Levasseur and Kaliaguine, 2008; Liu et al., 2018b; Liu et al., 2019) Mn^2+^ species were already present in the fresh Sm_0.96_MnO_3_ catalyst (Koch et al., 2020) balanced by about 5% Mn^4+^ resulting in an average oxidation state of 3.03. After heating in 20% O_2_, the average oxidation state decreased to 3.00. Switching to wet feed resulted in a further reduction. All Mn^4+^ vanished, and the fraction of Mn^2+^ increased to 4% balanced by Mn^3+^. While treating the sample in CO at 450°C, the Mn^2+^ fraction was growing to 5% leading to an average oxidation state of 2.95. Having switched back to wet feed, the Mn^2+^ fraction decreased to the level reached in the wet feed before the CO treatment at higher temperatures and dropped even further after switching back again to dry feed. Hence, the partial reduction of Mn^n+^ species at higher temperatures was caused by the gas phase composition and is a reversible phenomenon of the Sm_0.96_MnO_3_ perovskite catalyst (Koch et al., 2020).

**FIGURE 5:**
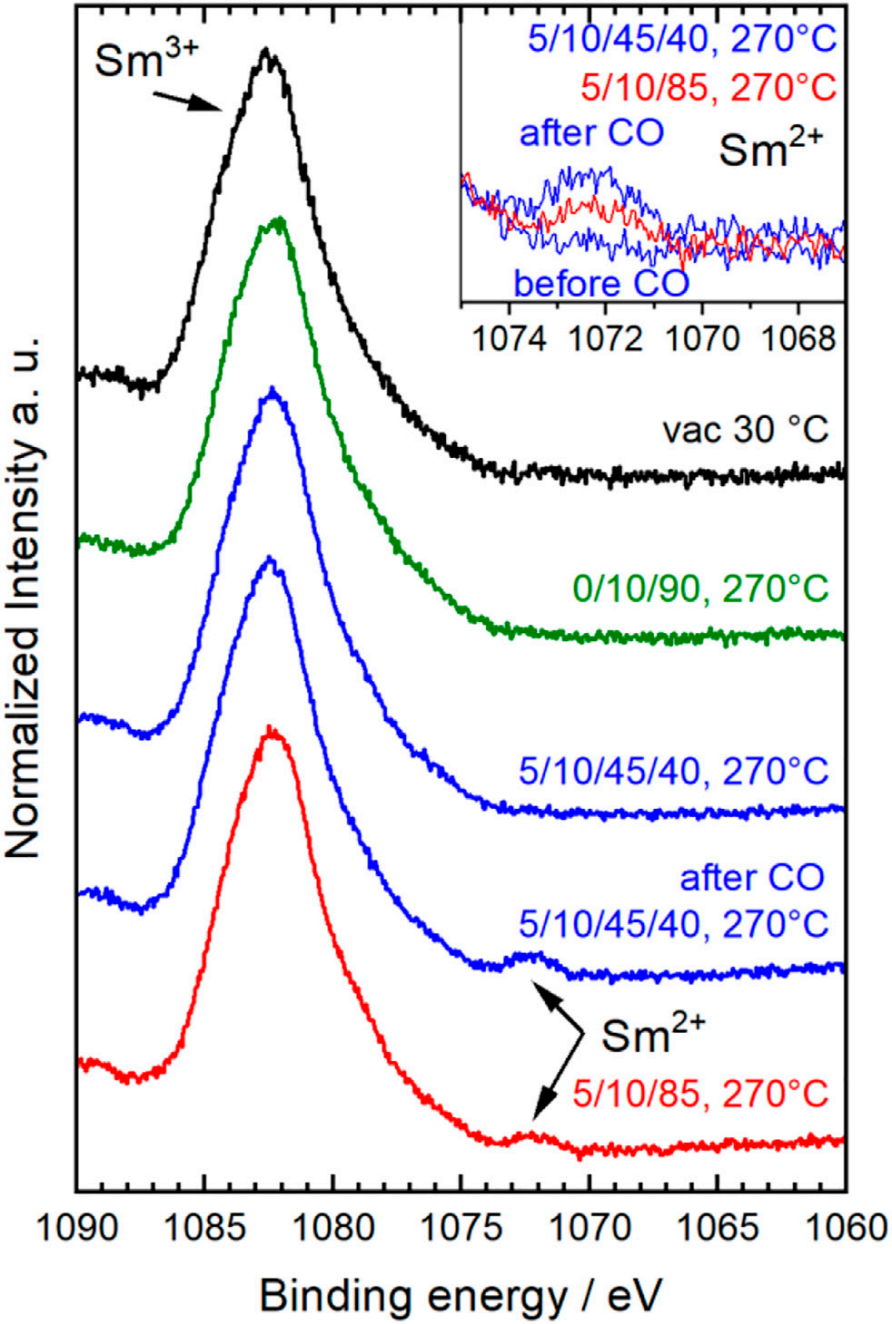
Normalized Sm 3d_5/2_ spectra of Sm_0.96_MnO_3_ collected at different conditions given as C_3_H_8_/O_2_/He/H_2_O (initial–black, 10% O_2_/He–green, wet feed–blue, dry feed–red). Inset depicts selected spectra ranges to show the Sm^2+^ peak.

Under the more reducing conditions in CO, also Sm^3+^ was slightly reduced indicated by a small peak at 1,072 eV in the Sm 3d_5/2_ spectrum (Figure 5) (Krill et al., 1980; Brunckova et al., 2019). This peak was observed after treatment in CO in wet feed and also still in the following dry feed (red spectrum in Figure 5). Its intensity decreased in dry feed compared to wet feed indicating that the process was reversible. But the reoxidation of the partially reduced A, i.e., Sm, was slow (Figure 5, inset) compared to the fast reoxidation of Mn^2+^. However, the reduction of Sm^3+^ was only detected by AP-XPS and not by AP-NEXAFS at the Sm M_5,4_-edges (Supplementary Figure S6) showing that only the outermost surface fraction of Sm^3+^ was reduced. Since the electron kinetic energy in the AP-XPS experiments was with 170 eV distinctly smaller compared to the primary kinetic energy of the electrons created in the XAS process at an excitation energy in the NEXAFS experiment (about 800 eV), the resulting depth of information was much smaller in the Sm 3d AP-XPS measurements compared to the AP-NEXAFS measurement at the Sm M-edges. In the NEXAFS spectra at the Sm M-edges, the small surface fraction of reduced Sm^2+^ species was apparently hidden by the larger fraction of Sm^3+^ species in the deeper layers.

**FIGURE 6:**
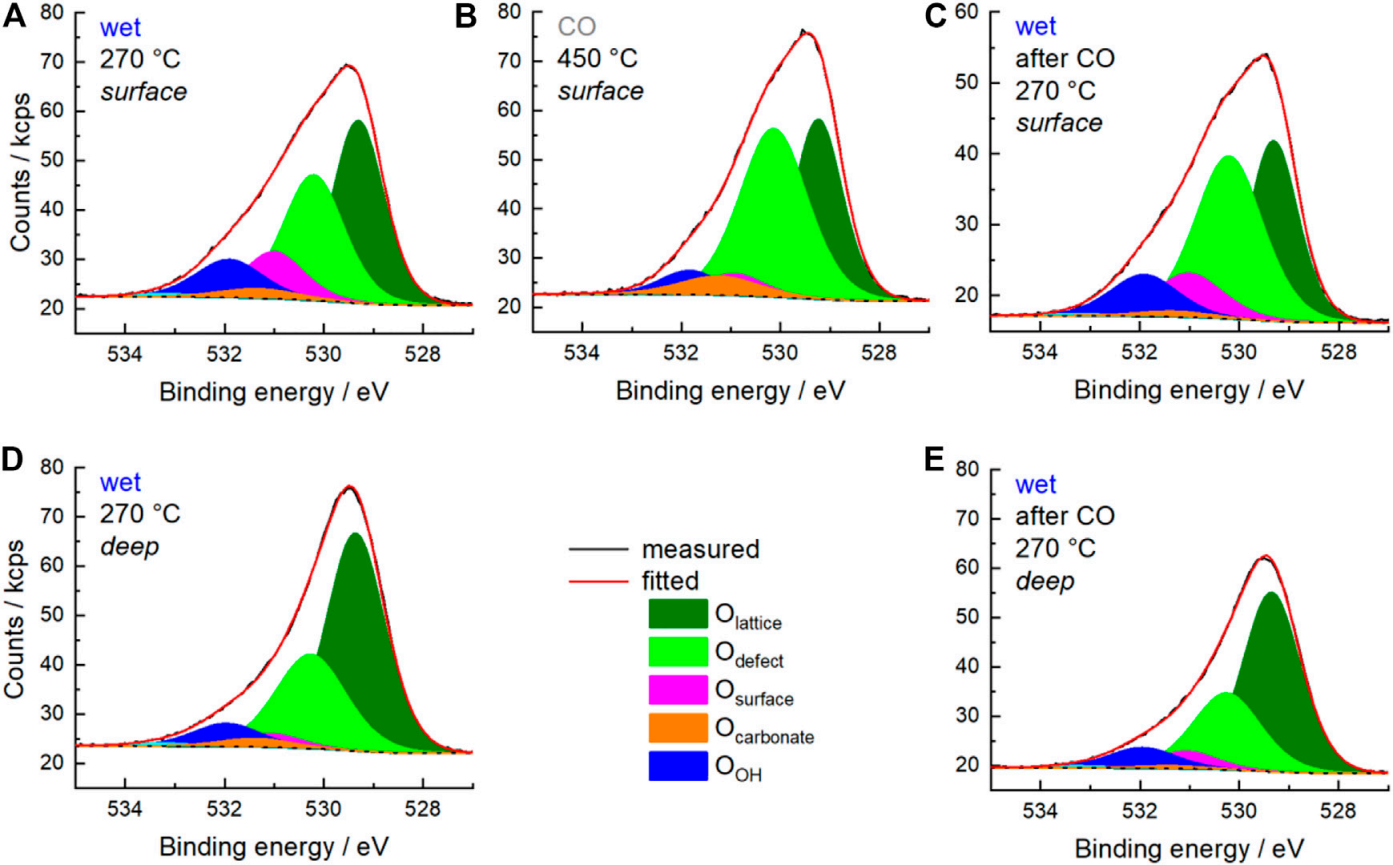
Measured O 1s spectrum (black), fitted spectrum (red) and background (black dots) measured in wet feed (C_3_H_8_/O_2_/He/H_2_O = 5/10/45/40) at 270°C **(A,C–E)** and in 25% CO balanced by He at 450°C **(B)**. Fitted peaks of O species are depicted as follows: O_lattice_ (olive) O_defect_ (green), O_surface_ (magenta), O_carbonate_ (orange), and O_OH_ (blue). **(A–C)** were measured with 700 eV excitation energy representing the surface region (*surface*). **(D,E)** were measured with 1,300 eV excitation energy representing information of deeper regions (*deep*).

**FIGURE 7:**
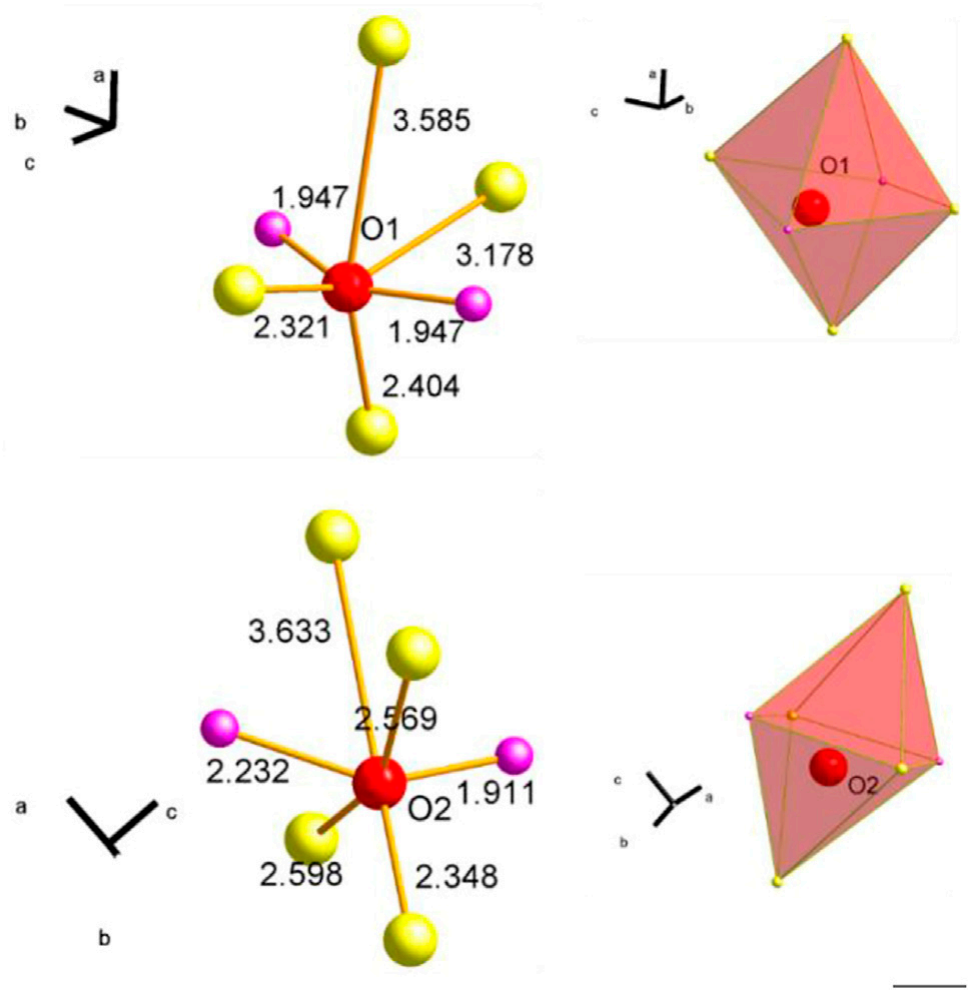
Representation of the two different lattice oxygen (red balls) O1 **(A)** and O2 **(B)** centered between next neighboring Mn atoms (purple balls) and Sm atoms (yellow balls). Numbers next to the illustrated connection between oxygen and metal atom (orange) give the distance between the atoms. Corresponding octahedral polyhedra are depicted on the right.

The partial reduction of Mn and Sm requires a charge compensation and the redox processes should be reflected in both the O 1s spectra and the O K-edge spectra. First, the O 1s spectra are discussed. In Figure 6 the measured O 1s and the corresponding fitted O 1s spectra are shown for the wet feeds and the CO treatment as surface scans (A), (B) and (C) as well as deep scans (D) and (E). Remarkably, the fraction of each oxygen species, which are introduced and discussed below, changes when comparing the outermost surface region (Figures 6A–C) with the deeper scans (Figures 6D,E). To describe all O 1s spectra measured under different conditions, different types of oxygen species should be considered according to the work published so far (Koch et al., 2020). The assignment and the fit model are summarized in Table 3. O_lattice_ represents the oxygen positioned in regular coordination in the crystal lattice. O_defect_ at 530.1 eV is oxygen, which is located in the vicinity of a defect (vacancy, off-centered position, hydroxyl group, etc.) (Fan and Goodenough, 1977; Sunding et al., 2011; Mierwaldt et al., 2014). Here it is noteworthy to mention that the oxygen atoms occupy two different positions in the orthorhombic lattice. Both oxygen atoms have each built up a distorted octahedral polyhedron consisting of two diametrically placed Mn atoms and four additional Sm atoms (Figure 7). These two octahedra differ slightly in their distortion. The distances between each oxygen position and its metal neighbors vary slightly, but the average distances between oxygen atoms and Mn and Sm atoms are similar (Supplementary Table S2). Since the two different oxygen species each have a different local environment, they should be spectroscopically distinguishable. However, it is not clear whether these differences can be resolved experimentally. This requires further experiments and the support of theory. From the available experiments, there is no clear evidence to further distinguish between an O_lattice1_ and an O_lattice2_, which lay below the signal of O_lattice_ and could also overlap with the signal of O_defect_. Therefore, according to the literature, only the O_lattice_ and O_defect_ species are considered to describe the volume of the perovskite in the following discussion. The species O_surface_ at 530.9 eV is attributed to an electrophilic oxygen species adsorbed on the surface (Merkle and Maier, 2008; Mierwaldt et al., 2014; Stoerzinger et al., 2014). The binding energy 531.2 eV was uniquely assigned to O_carbonate_ because the peak intensity of this signal correlates with the peak intensity of C_carbonate_ from C 1s spectra at a binding energy of 288–289 eV (Stoerzinger et al., 2014; Koch et al., 2020). In addition, O_OH_ species are assigned to the binding energy at 531.8 eV (Nesbitt and Banerjee, 1998; Banger et al., 2011). Adsorbed H_2_O and adsorbed oxygenates at binding energies of 532.2 eV and 534–535 eV, respectively, are present at higher temperatures only with a fraction of less than 1%.

**TABLE 3:** Energy positions (BE) of oxygen species in O 1s spectra and applied peak width (FWHM) for fitting all O 1s spectra (Koch et al., 2020).

Oxygen species	O_lattice_	O_defect_	O_surface_	O_carbonate_	O_OH_	O_H2O__ads_	O_oxygenates ads_
BE/eV	529.2	530.1	530.9	531.2	531.8	533.2	534–535
FWHM/eV	1.1–1.3	1.4–1.6	1.4–1.6	1.7–2.0	1.4–1.6	1.5–1.7	1.5–1.7

**FIGURE 8:**
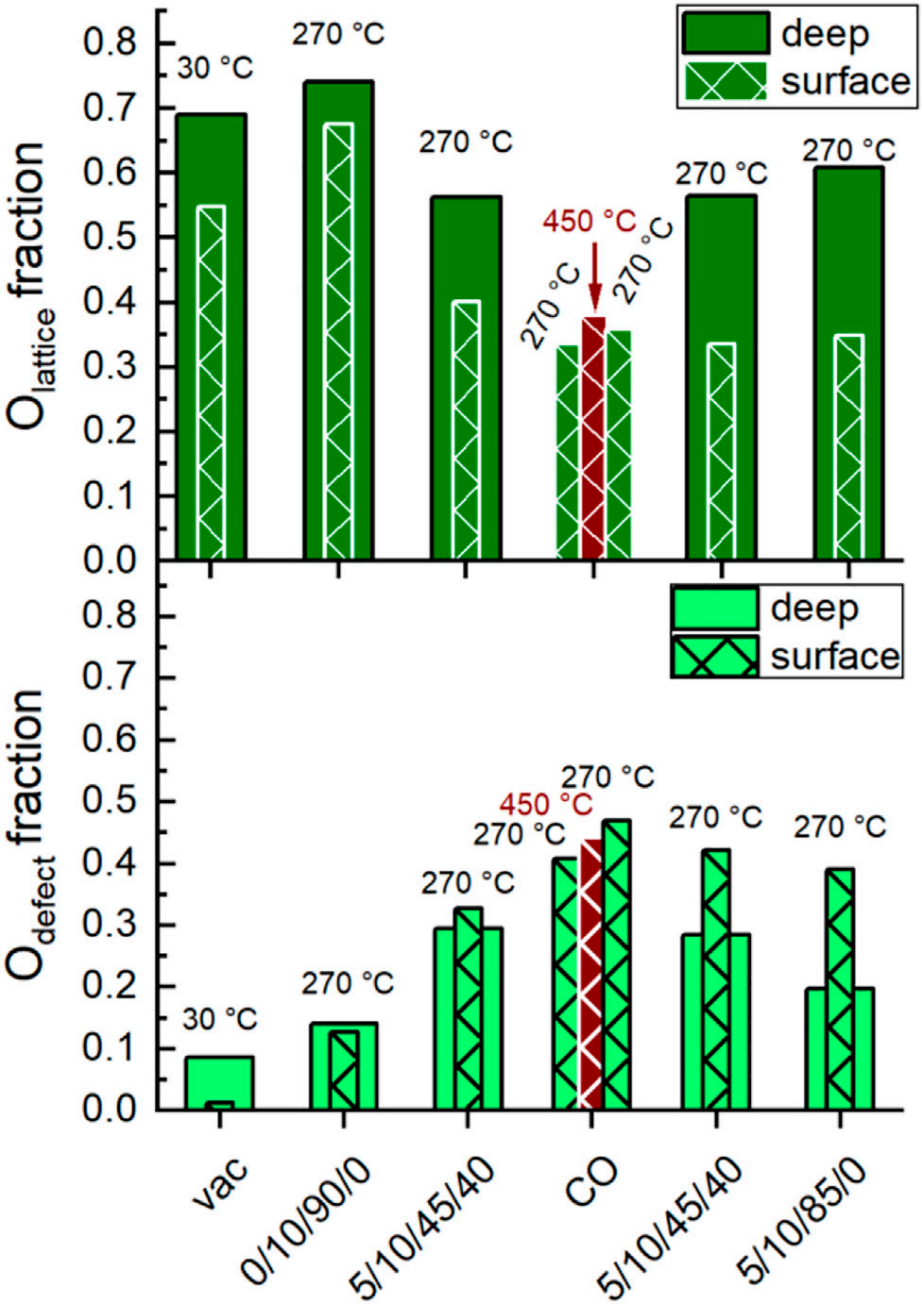
Evolution of the fraction of O_lattice_ (top) and O_defect_ (bottom) at different temperatures and feeds (C_3_H_8_/O_2_/He/H_2_O) given below. Deep, filled columns: Extracted from O 1s peak fitting of spectra conducted using 1,300 eV excitation energy. Surface, hatched columns: Extracted from O 1s peak fitting of spectra conducted using 700 eV excitation energy. Dark red, surface, hatched columns: Extracted from O 1s peak fitting of spectra conducted at 450°C using 700 eV excitation energy.

**FIGURE 9:**
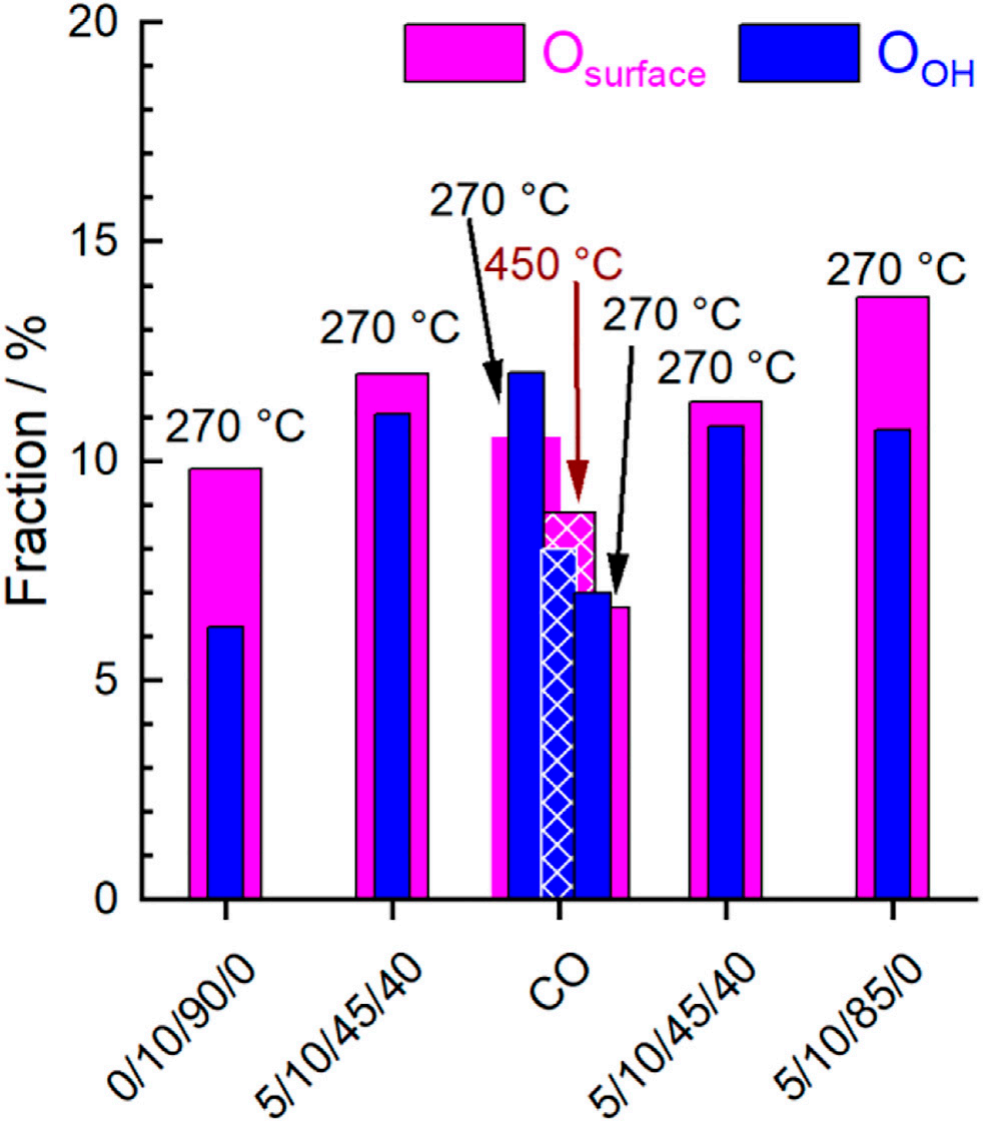
Evolution of the fraction of O_surface_ (magenta) and O_OH_ (blue) extracted from O 1s peak fitting at different temperatures and feeds (C_3_H_8_/O_2_/He/H_2_O) given below. Hatched columns represent fractions at 450°C, filled columns represent fractions at 270°C.

Figure 8 and Figure 9 show the evolution of the fraction of oxygen species as a function of the conditions applied. Two photon energies were used for measuring the O 1s spectra. At 700 eV (inelastic mean free path IMFP = 0.6 nm), mainly the outermost surface is analyzed. At 1,300 eV (IMFP = 1.6 nm), deeper surface layers are also detected. In Figure 8 the depth profile for O_lattice_ and O_defect_ is shown. In general, the amount of O_lattice_ was larger in deeper layers than at the outermost surface. With increasing temperature, the fraction of O_defect_ increased, especially at the outermost surface (Figure 8), which supports the plausibility of the applied fit model.

After heating up in synthetic air to 270°C and subsequently reducing the oxygen content from 20 to 10%, the surface of the catalyst was healed and exhibited the largest fraction of O_lattice_. Minor fractions of O_defect_, O_surface_, and O_OH_ were also observed indicating complex surface structures. Carbonates were already largely decomposed at the reaction temperature, so their contribution to the O 1s spectrum could be neglected. Remarkably, the increase in O_defect_ fraction after heating indicates that defect-rich surface structures have already been generated by the increased temperature and/or reduced oxygen partial pressure. This is consistent with model calculations that have shown that defect-rich surfaces of LaMnO_3_ are energetically favored (Evarestov et al., 2003; Evarestov et al., 2004). The change in chemical potential by switching to wet feed caused an increase in the concentration of O_defect_, O_OH_ and O_surface_ at the expense of O_lattice_ (Figure 8 and Figure 9). Hence, either the H_2_O in the feed or the alkane and its derivatives or both actively modified the surface of the catalyst. After treatment with CO at 450°C and decreasing the temperature in CO to 270°C, a further little decrease of O_lattice_ and a distinct decrease of O_surface_ and O_OH_ was observed. At the same time, the fraction of O_defect_ increased. This is consistent with the partial reduction of Mn^3+^ and Sm^3+^ observed using AP-NEXAFS and AP-XPS, respectively (Figure 4 and Figure 5). The surface oxygen vacancies could have been formed by the oxidation of CO to CO_2_ using surface oxygen as an oxidant (Hui et al., 1993). This was confirmed by repeating the CO treatment in a thermogravimetry experiment using a mass spectrometer for gas analysis. Only the formation of CO_2_ was observed while heating up to 450°C in 2% CO balanced by Ar (Supplementary Figure S7). Switching to the wet reaction feed caused an increase in O_surface_ and O_OH_ and a simultaneous slight decrease in O_defect_. The fraction of O_lattice_ at the outermost surface remained unchanged. Comparing the wet feed before CO treatment with the wet feed after CO treatment, the amounts of O_surface_ and O_lattice_ in the surface region were slightly lower after CO treatment, but the O_defect_ concentration was clearly higher, while the amount of O_OH_ remained similar (Figure 8 and Figure 9). Thus, the distribution of oxygen species in the surface region resembled the state before CO treatment, except for O_defect_ species, which was present in higher concentrations. The higher concentration of O_defect_ is supported by the thermogravimetry in CO-containing atmosphere. The found mass loss of about 0.6% corresponds theoretically to the removal of all oxygen atoms linked to a Mn or a Sm of the outermost surface layer (Supplementary Figure S8, right). This would imply the reduction of the surface to the metallic state by removal of all oxygen atoms of the outermost surface layer (Supplementary Figure S8, left), which is not found spectroscopically. Only 0.2% mass loss is estimated assuming a 001 surface showing a balanced A/B ratio as found for this catalyst (Table 2) and assuming fully saturated with oxygen atoms (Supplementary Figure S8, left). This means also oxygen from the bulk or at least from the near-surface range reacts with CO. Hence, more oxygen vacancies are likely formed and thus the number of O_defect_ increases, which is oxygen in the vicinity of a defective site. By removing H_2_O from the feed, the O_lattice_ fraction in the deep and surface areas increased slightly again (Figure 8). At the same time, the proportion of O_defect_ on the surface decreased slightly. This decrease was more evident in the deep region, indicating an ongoing healing process in the bulk. Moreover, the amount of O_surface_ increased and the amount of O_OH_ slightly decreased (Figure 9). According to data measured in this study, O_surface_ appears to be required to start the healing process, reduce the amount of O_defect_ and increase the amount of O_lattice_ again.

**FIGURE 10:**
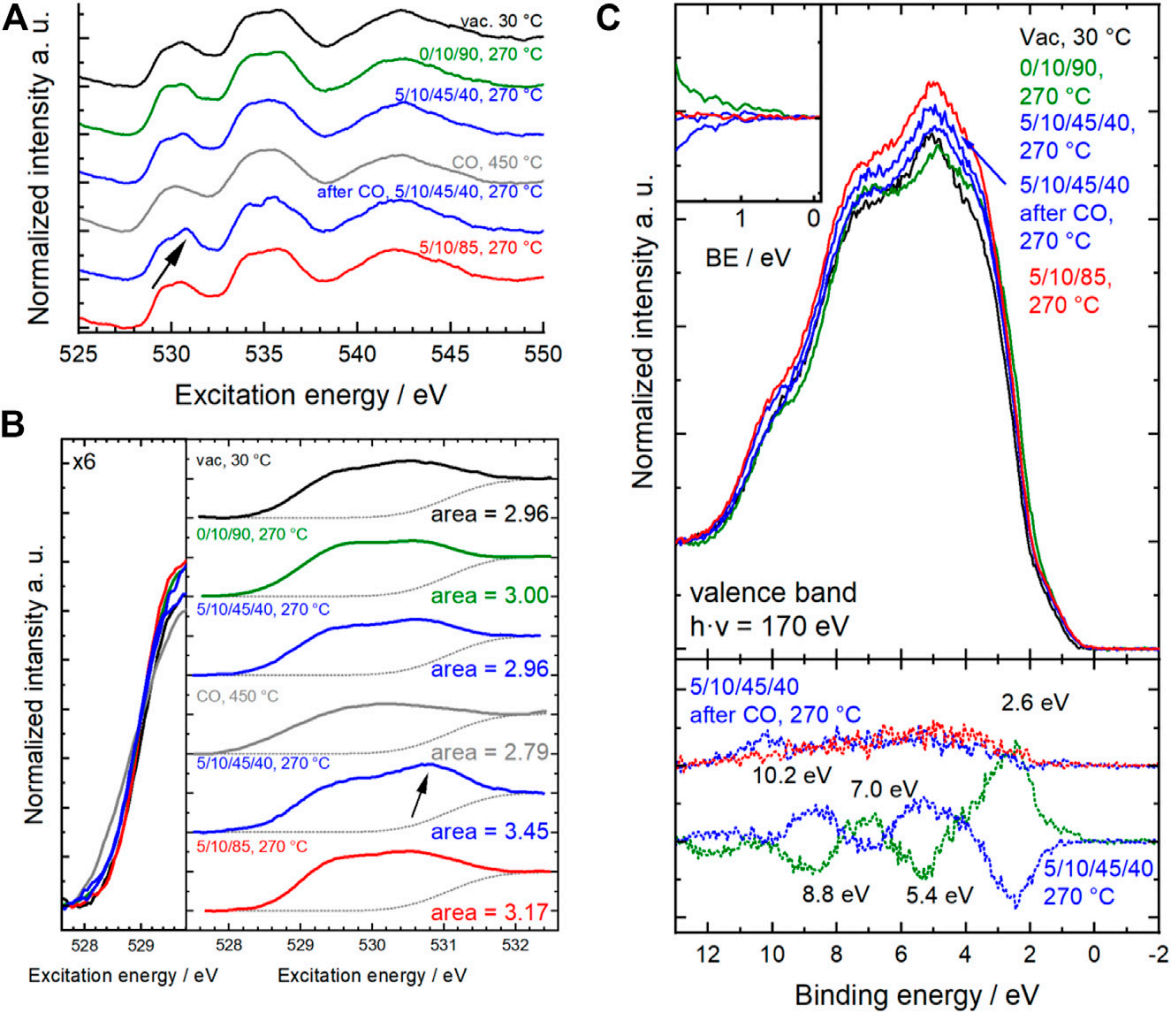
**(A)** Normalized O K-edge spectra measured in different atmospheres and temperatures. **(B)** Corresponding pre-edge range of O K-edges. Left: enlarged overlay at pre-peak onset. Right: Pre-peak including the step function in grey dots representing the background. Resulting areas of pre-edge peak are given on the right. Arrows in **(A,B)** indicate the same position of most changing range in the pre-edge region. **(C)** Corresponding normalized valence band spectra measured in different atmospheres and temperatures. In addition, difference spectra are plotted in dots at the bottom. Enlarged difference spectra are depicted in the inset at the top left. Difference spectra show the difference to previous conditions. Conditions are given as C_3_H_8_/O_2_/He/H_2_O (initial–black, 10% O_2_/He–green, wet feed–blue, CO/He–grey, dry feed–red).

Surface modifications and the increase of O_defect_ species were confirmed by near-edge X-ray absorption spectroscopy at the O K-edge (Figures 10A,B). In transition metal oxides containing 3d B metals, the first absorption above the threshold at ∼530 eV corresponds to the excitation from the O 1s state to the hybridized O 2p-B 3d state. The spectrum is difficult to interpret because the intensity and shape of this feature are related in a complex way to a multitude of overlapping and interconnected phenomena, such as oxidation state, spin state, degree of hybridization, strain and defects in the oxide structure. The 3d band of Sm_0.96_MnO_3_ is split into two peaks at 529.5 and 530.8 eV, which are in the simplified picture of the ligand field theory assigned to Mn e_g_ and t_2g_ states, respectively (Mierwaldt et al., 2014; Frati et al., 2020). In the spectra shown here (Figures 10A,B) the positions match well to those reported. The e_g_ peak is invariant at 529.5 eV while the t_2g_ peak maximum shifts from 530.5 eV in dry feed to 530.7 eV in wet feed after CO treatment. When the chemical potential of the atmosphere was changed, a change in the onset (during CO treatment at 450°C, grey spectrum) and intensity of the pre-edge peak was clearly observed (Figure 10B left), indicating that charge transfer had occurred. Also, the shape of the peak changed drastically. The overall area of the pre-peak was integrated taking into account a step function for the ionization to the continuum (Figure 10B right). The area below the pre-peak ranges from 2.79 during CO treatment to 3.45 in wet feed after CO treatment. The O K-edge after the CO treatment in wet feed differs strongly from those measured in the wet feed before the CO treatment. Since the pre-edge peak represents holes in the band, the number of holes is consequently reduced when the catalyst is reduced during CO treatment, resulting in a small area of 2.79. Switching back to wet feed yields in a significant increase in the area to 3.45 which is clearly visible from the increased t_2g_ peak at 530.7 eV (Figures 10A,B arrow). This number is also higher compared to 2.96 measured in the wet feed before CO treatment. Hence, the CO treatment tremendously modified the surface and the sub-surface of the catalysts in the way that the CO reduced partially the surface metals Mn and Sm (Figure 4 and Figure 5) and generated more O_defects_ (Figure 8), which is supported by thermogravimetry measurement. Finally, these surface modifications yielded in an increased number of holes in the t_2g_ band when the water of the wet feed or one of the other feed ingredients or reaction intermediates come into play. After switching to the dry feed, the area below the pre-peak is with 3.17 still increased compared to 2.96 in the wet feed before, but it already indicates the slow healing.

The indication of partial reduction of Mn^3+^ to Mn^2+^ in CO at 450°C was complementarily confirmed by Mn L_2,3_-edge spectra (Figure 4) and valence band spectroscopy (Figure 10C). In addition, adsorption of propane and propene and the formation of OH groups were evidenced by the appearance of peaks at 8.8 and 10.2 eV (Wernbacher et al., 2019). Thus, the reactants are not only converted on the catalyst surface but also modify the catalyst surface, which results in a reactant-dependent catalytic behavior.

## Discussion

The optimized combustion method is suitable to achieve batches in gram scale of phase pure Sm_0.96_MnO_3_ powders having low bulk density due to the agglomerated crystallites forming caves, hollow spheres, and large networks (Supplementary Figure S3). The in this way prepared Sm_0.96_MnO_3_ can be operated as a catalyst in the oxidative dehydrogenation of propane (Koch et al., 2020). The Sm_0.96_MnO_3_ is characterized by a stable bulk crystal structure under the applied reaction conditions. Neither crystallite growth (Table 1) nor lattice expansion, which were observed for similar LaMnO_3_ perovskites (Miyoshi et al., 2003; Koch et al., 2020) occurred. Both show the stability of the lattice and imply that extensive re-organization of the lattice can be neglected.

**FIGURE 11:**
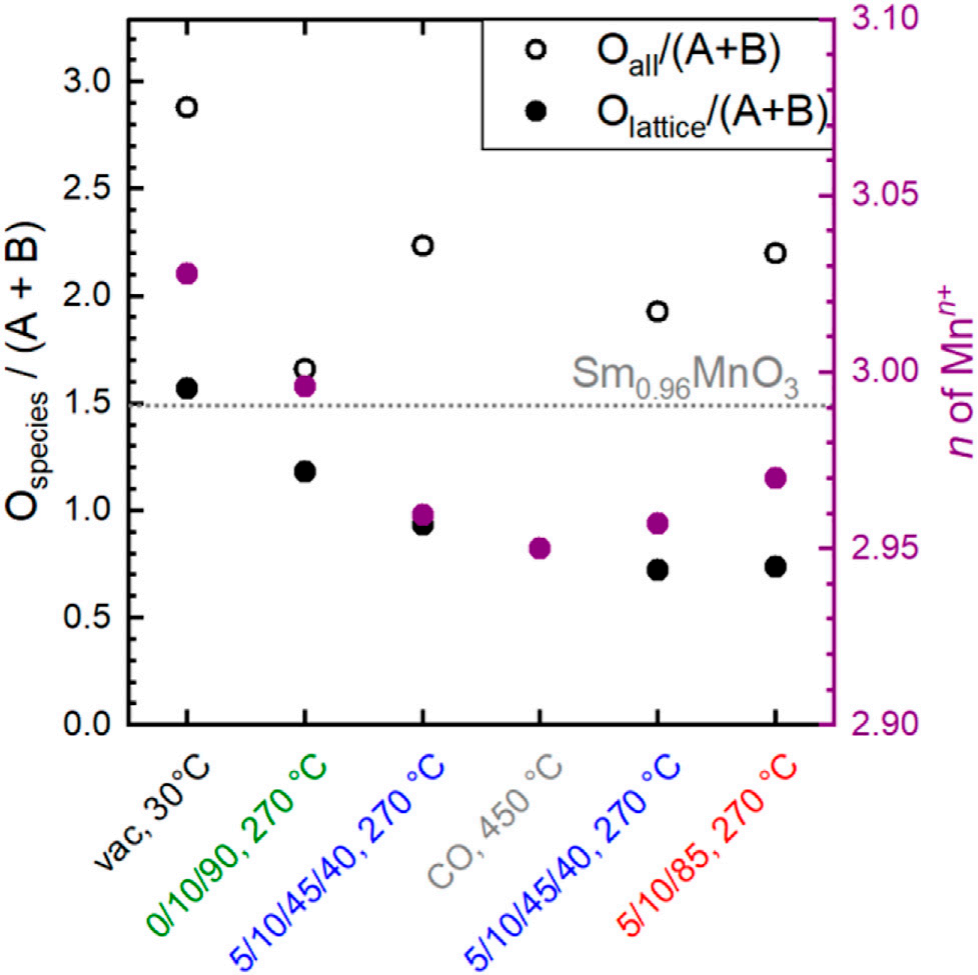
The ratio of the sum of all fitted oxygen (O_all_) species and the sum of Mn + Sm obtained from XPS analysis (empty circles) in dependence on conditions applied and the ratio of the amount of O_lattice_ and the sum of Mn + Sm obtained from XPS analysis (full circles) in dependence on conditions applied. Purple full circles depict the Mn oxidation state obtained from Mn L-edge fitting. C_3_H_8_/O_2_/inert = 5/10/85 (dry); C_3_H_8_/O_2_/inert/H_2_O = 5/10/45/40 (wet). The dotted line represents the number from bulk analysis for comparison (XRF and O content).

However, the functionality of the catalyst changes in terms of both activity and selectivity when different pretreatments and wet feed are employed showing that the chemical potential affects the surface of the catalyst and influences, thus, the catalytic properties. The chemical potential reversibly modifies the oxidation state of metals in the surface region. Even heating to 270°C in an oxygen-containing atmosphere and then switching to wet feed leads to full reduction of all initially measured Mn^4+^ species to Mn^3+^ and also to an increased formation of Mn^2+^. This illustrates the importance of operando techniques, which can reveal the dynamic redox behavior of the Sm_0.96_MnO_3_ catalyst under various conditions. The partial reduction of Mn^n+^ in perovskites, e.g. in the oxygen evolution reaction (Mierwaldt et al., 2014) or the oxidative dehydrogenation of propane (Koch et al., 2020) has been related to H_2_O in the atmosphere and is feasible when Mn^n+^ occupies an A position (Ignatans et al., 2019; Koch et al., 2020) in the ABO_3_ perovskite structure. In the A-deficient Sm_0.96_MnO_3_ catalyst prepared here, some small Mn^n+^ ions probably sit on spacious A positions. The changes in oxidation state are balanced by types and amount of oxygen in the surface region. When the Mn is partially reduced, also the amount of O_lattice_/(Sm + Mn) is reduced showing that the partial reduction is accompanied by loss of oxygen atoms sitting on a perfect lattice position (Figure 11 grey and purple circles). Thus, the increase in O_defect_ is expected, which supports the applied fit model. In general, more oxygen (O_all_ is the sum of all species) was found using AP-XPS (1.66 < O_all_/(Sm + Mn) < 2.98) than with the chemical analysis of the bulk (O_bulk_/(Sm + Mn) = 1.49), showing a highly defective surface. The chemical analysis cannot distinguish between oxygen in different configurations (e.g., O_lattice_, O_defect_), but gives just the sum O_bulk_ (oxygen determined by bulk chemical analysis). The O_lattice_ content determined by surface analysis varies as a function of conditions as follows: 0.72 < O_lattice_/(Sm + Mn) < 1.56. It decreases to a minimum in the wet feed after treatment in CO at 450°C and slowly increases in the subsequent dry feed again (Figure 11). Hence, the amount of oxygen in the surface region in relation to A and B does not only depend on the type of B as previously discussed (Fierro and Tejuca, 1987) but also on the feed and the temperature. Interestingly, when only oxygen was present in the atmosphere at 270°C, bulk and surface analysis yielded the most similar figures for O_all_/(Sm + Mn), namely 1.49 and 1.66, respectively, with O_lattice_ having the largest fraction in the surface analysis. This was larger here than in all other conditions. However, the crystal lattice was not perfect, as the O_lattice_/(A + B) ratio of 1.18 in the outmost surface layer was lower than the expected ratio of 1.49 for a perfect Sm_0.96_MnO_3_ lattice (Figure 11, dotted line). This is because there are always unsaturated atoms on the surface. Therefore, only a perfect, defect-free lattice would result likely in a consistent bulk and surface composition. Conversely, it can be deduced that the over-stoichiometry of oxygen in the surface region already indicates additional oxygen species, with the interaction of this oxygen with the metals being lower than the average M-O interaction in the bulk. Noteworthy to mention, the additional oxygen is not coming simply from adsorbed H_2_O wetting the surface because the corresponding water signal at 533.2 eV (Table 3) decreases after heating to 270°C in all atmospheres due to high temperature (Figure 8). The oxygen excess on the surface is based on O_OH_, O_surface_, a little amount of O_carbonate_ and O_defect_. Especially, the O_defect_ is the major non-lattice oxygen species. The amount of O_defect_ is increased in wet feed (Mierwaldt et al., 2014; Koch et al., 2020) and further increased by reductive treatment in CO. It slowly decreases in the wet feed again and decreases, even more, when dry feed is employed (Figure 8). Simultaneously, Mn and Sm are partially reduced and reoxidized, respectively. Moreover, the O_defect_ increases apparently at the expense of O_lattice_ when the reaction feed streams over the catalyst (Figure 8). The oxygen can undergo hydrogenation through propane and its derivatives creating thus an O_OH_, also indicated in the difference plots of the valence bands (Figure 10C), when the oxygen is bound to an Mn (Li et al., 2019). In vicinity to this *in situ* formed hydroxyl group also O_defects_ are created causing thus the increase in surface concentration of O_defect_ species in the feed. These findings suggest that the creation of the O_defect_ is related to both hydroxylation and oxygen vacancy formation. Moreover, the peak increase in the pre-peak region of the O K-edge NEXAFS spectra at 530.8 eV in wet feed underlines the formation of defective structures. This is also reflected in the valence band measurements, which provide information on the occupied states (Figure 10C).

In conclusion, the assignment of the O 1s signal at 530.1 eV to oxygen, which is part of irregular surface structures (O_defect_), is strongly supported by simultaneously observed charge transfer in the O K-edge spectra and the valence band spectra. Hence, O_defect_ represents the defective state of the surface and this defective perovskite surface catalyzes the oxidative dehydrogenation of propane more to propene compared to a less defective perovskite surface. Once the healing is finished, the initial catalytic properties of Sm_0.96_MnO_3_ are recovered, which is reflected in the similar performance in the dry feed after the different pretreatments (Figure 1A). Moreover, remembering that Mn^3+^ is partially reduced in wet feed but simultaneously more holes are observed indicating reactant-induced charge distribution in the outermost surface region. Therefore, the healing cannot be easily described as the filling of formed oxygen vacancies. Many processes cause charge transfer and may thus contribute to the catalytic properties of the Sm_0.96_MnO_3_ catalyst in oxidative dehydrogenation of propane.

**FIGURE 12:**
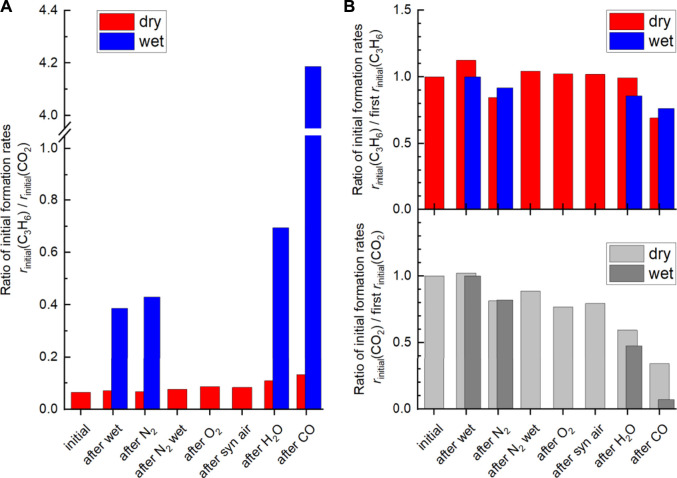
**(A)** Ratio of initial propene formation rate and CO_2_ formation rate in dry feed (red) and wet feed (blue). **(B)** Ratio of initial propene (C_3_H_6_) formation rates (r_initial_ (C_3_H_6_)) after the different treatments and initial propene formation rate at the beginning of the experiment (condition: initial in dry feed (red) and in wet feed (blue) on the top. The ratio of initial CO_2_ formation rates after the different treatments and initial CO_2_ formation rate (r_initial_ (CO_2_)) at the beginning of the experiment (condition: initial in dry feed (light grey) and wet feed (dark grey) on the bottom. Data obtained from contact time variation at 300°C. Contact time (W/F) ranges from 0.00347 g·min·L^−1^ to 0.00947 g·min·L^−1^ in dry feed (C_3_H_8_/O_2_/inert = 5/10/85) and wet feed (C_3_H_8_/O_2_/inert/H_2_O = 5/10/45/40).

**FIGURE 13:**
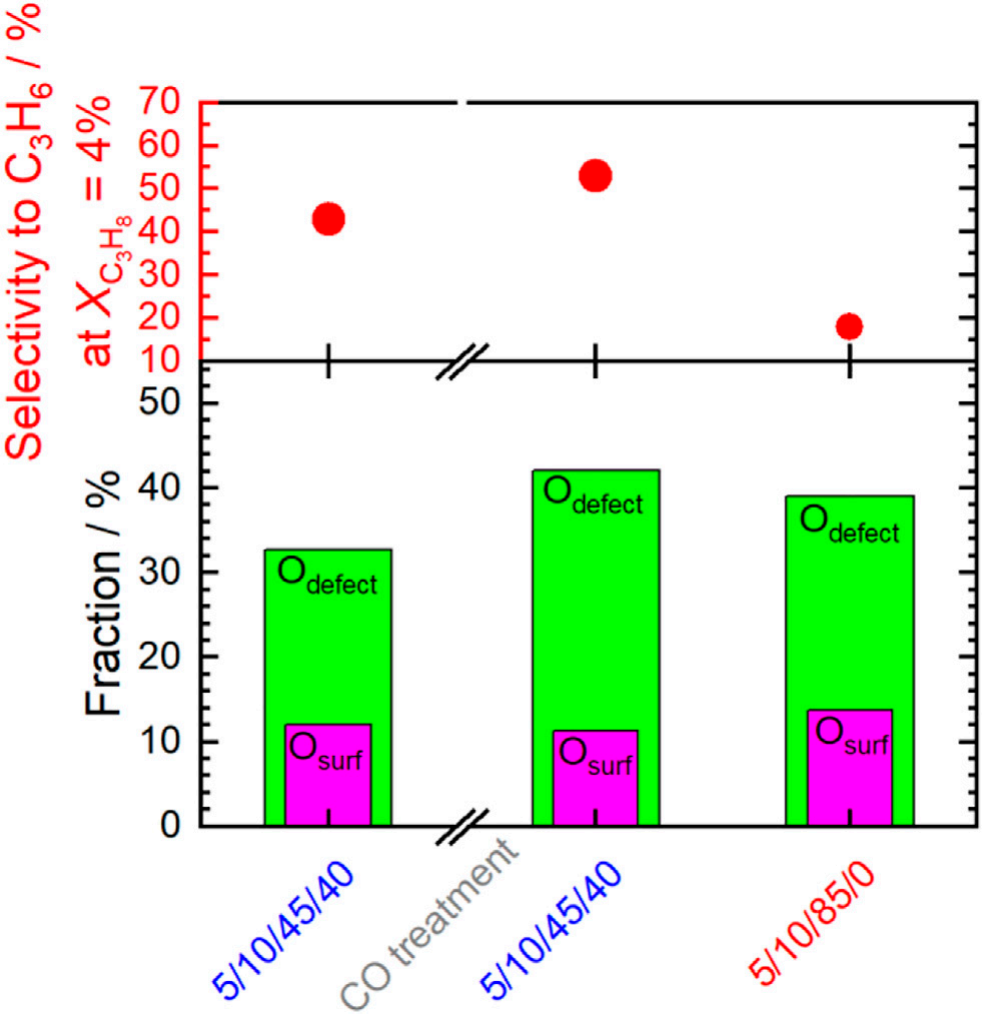
Selectivity to propene at 4% conversion of propane in wet feed (5/10/45/40), in wet feed after CO treatment and in dry feed (5/10/85/0) in the top panel. Corresponding fractions of O_defect_ and O_surface_ are plotted in the bottom panel.

Remarkably, switching from dry to wet feed results in an increase in the selectivity to propene. This is more pronounced after additional treatment at higher temperatures and more reducing chemical potentials. Moreover, it is reversible after switching to dry feed. The products formed in propane oxidation are only propene and CO_2_. This implies that possibly formed intermediates are fully oxidized under the conditions applied. Changes in selectivity can be caused by either enhanced formation of propene or by reduced formation of CO_2_. In order to compare the intrinsic selectivity of the catalyst after various treatments, the initial formation rates of propene and CO_2_ are compared (rates extrapolated to 0% conversion). The ratio between the initial propene formation rate and the initial CO_2_ formation rate is depicted in Figure 12A. The ratio is almost independent of the pretreatment when the dry feed is set. It slightly increases after steaming and CO treatment (red columns). In contrast to the dry feed, the ratio distinctly increases when the wet feed is employed. In addition, steaming and even more CO treatment cause a further distinct increase of the ratio. Whether the distinct improvements in the wet feed are more related to the increase in propene formation rate or by reduced CO_2_ formation rate becomes apparent when the formation rate of each product, i.e., propene or CO_2_, after respective treatment are compared to the initially measured formation rate of each product before pretreatments (Figure 12B). The loss in activity after treatments (Figure 1) is clearly mirrored in the loss of the CO_2_ formation rate. The corresponding ratio of the initial CO_2_ formation rate after each treatment and the initially measured CO_2_ formation rate (Figure 12, “initial”) decreases accordingly. The decrease of the CO_2_ formation rate appears significantly after treatment in steam and even more in CO. In particular, the CO_2_ formation rate strongly decreases in the wet feed. Therefore, treating the catalyst with reducing atmospheres results mainly in the loss of formation rate of the total combustion product CO_2_. Presumably, the surface structure responsible for total oxidation is degraded by the treatments, because the feed was the same before and after treatments and thus their effects related to the reactants can be excluded. However, at higher conversions of propane, the performance of the catalyst approaches to those initially measured suggesting that the structures required for total oxidation are slowly recovered. It still cannot be deduced whether the CO_2_ which is less formed due to the treatment originates from direct oxidation of propane or oxidation of intermediately formed propene. However, the selectivity changes could indicate an important role of oxygen on perovskite catalysts in propane oxidation. Thus, O_surface_ species could be responsible for both the high activity and the high total oxidation yield. These oxygen species are present in all conditions applied. Although the selectivity to propene is improved by reductive treatments in wet feed, CO_2_ formation cannot be fully suppressed. However, the CO_2_ formation rate increases when switching from the wet feed to the dry feed, which is in agreement with the observed decreased selectivity to propene and thus to increased total oxidation. In this discussion, it is assumed that the changes in selectivity are governed by the changes in CO_2_ formation rate while the apparent formation rate of propene varies little (Figure 12B). The O_surface_ species might be more important than O_OH_ and O_defect_ in terms of selectivity, which explains the lower selectivity to propene in dry feed, although more O_defect_ was measured here (Figure 8 and Figure 9). The O_surface_ species could also be required for the healing of the oxygen vacancies and thus reduction of O_defect_ species, which have been created by treatments at higher temperatures. This is shown in Figure 13. The selectivity to propene increases after CO treatment with increasing proportion of O_defect_, but decreases again after switching to dry feed, because O_defect_ decreases and O_surface_ increases. The concentration of surface defects and the concentration of electrophilic oxygen on the surface are interrelated and also have opposite and simultaneous effects on the selectivity to propene, which makes direct correlations difficult.

In summary, it can be concluded that the catalyst is stable in its bulk structure but the surface changes significantly under propane oxidation conditions as a function of the feed applied and thus as a function of the chemical potential. The local geometry of the terminating atoms changes and is limited to the outermost surface of the catalyst. These changes are clearly reflected in the core level and valence band spectra, as well as in the X-ray absorption spectra. However, in total these structural surface transformations are of minor extent. Therefore, the positive effect on the selectivity in propane oxidation is only slight and only significant at low conversion rates. But these results clearly show that for the optimization of a catalyst, not only its solid state and surface chemistry must be known, but also the kinetics of the chemical reactions in the solid state under different process conditions. In the search for more selective catalysts, other methods of catalyst synthesis should be used that more effectively increase the number of defects and thus the dynamic transformation of the catalytically active surface structures.

## Data Availability

The raw data supporting the conclusion of this article will be made available by the authors, without undue reservation.
