# Supplementary material for: The Influence of the Chemical Potential on Defects and Function of Perovskites in Catalysis
Source: Front Chem. 2021 Sep 17;9:746229. doi: 10.3389/fchem.2021.746229 (PMC8485044; doi:10.3389/fchem.2021.746229)
Supplement: Supplementary file 1 [file DataSheet1.docx]

Supplementary Material

The Influence of the Chemical Potential on Defects and Function of Perovskites in Catalysis

Gregor Koch^1^, Michael Hävecker^2^, Pierre Kube^1^, Andrey Tarasov^1^, Robert Schlögl^1,2^,
Annette Trunschke^1*^

^1^Fritz-Haber-Institut, Department of Inorganic Chemistry, Max Planck Society, Berlin, Germany

^2^Max Planck Institute for Chemical Energy Conversion, Heterogeneous Reactions, Max Planck Society, Mühlheim, Germany

*** Correspondence:**Annette Trunschke
trunschke@fhi.mpg.de

# Supplementary Figures and Tables

## Supplementary Figures


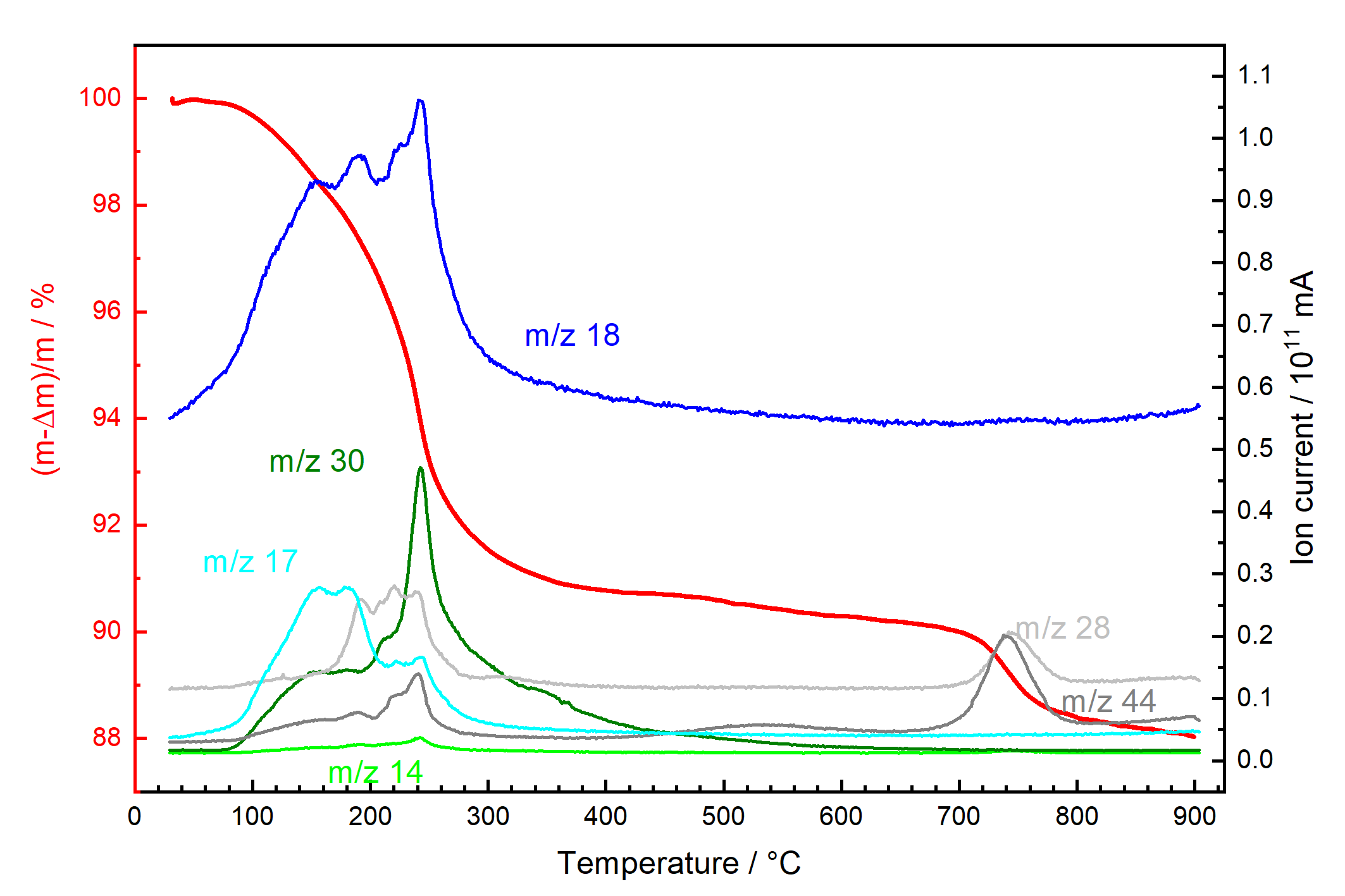


Supplementary Figure S1 Mass loss of the combustion product in combination with the most intense ionic currents of decomposition products: m/z = 17 H_2_O, amines from glycine, m/z = 18 H_2_O, ^18^O from fragments of glycine, CO_2_ and NO, m/z = 28, 44 fragments of glycine, CO_2_, m/z = 14, 30 NO measured by TG-MS. No decomposition products were detected at temperatures higher than 800 °C. The sample was heated at 10 °C∙min^-1^ to 900 °C while 70 ml∙min^-1^ of a gas mixture (21% O_2_ balanced by Ar) streamed over the sample.





Supplementary Figure S2 Diffraction patterns of the fresh (A) and the used (B) catalyst including the fitted patterns (red) and the corresponding difference patterns (blue).


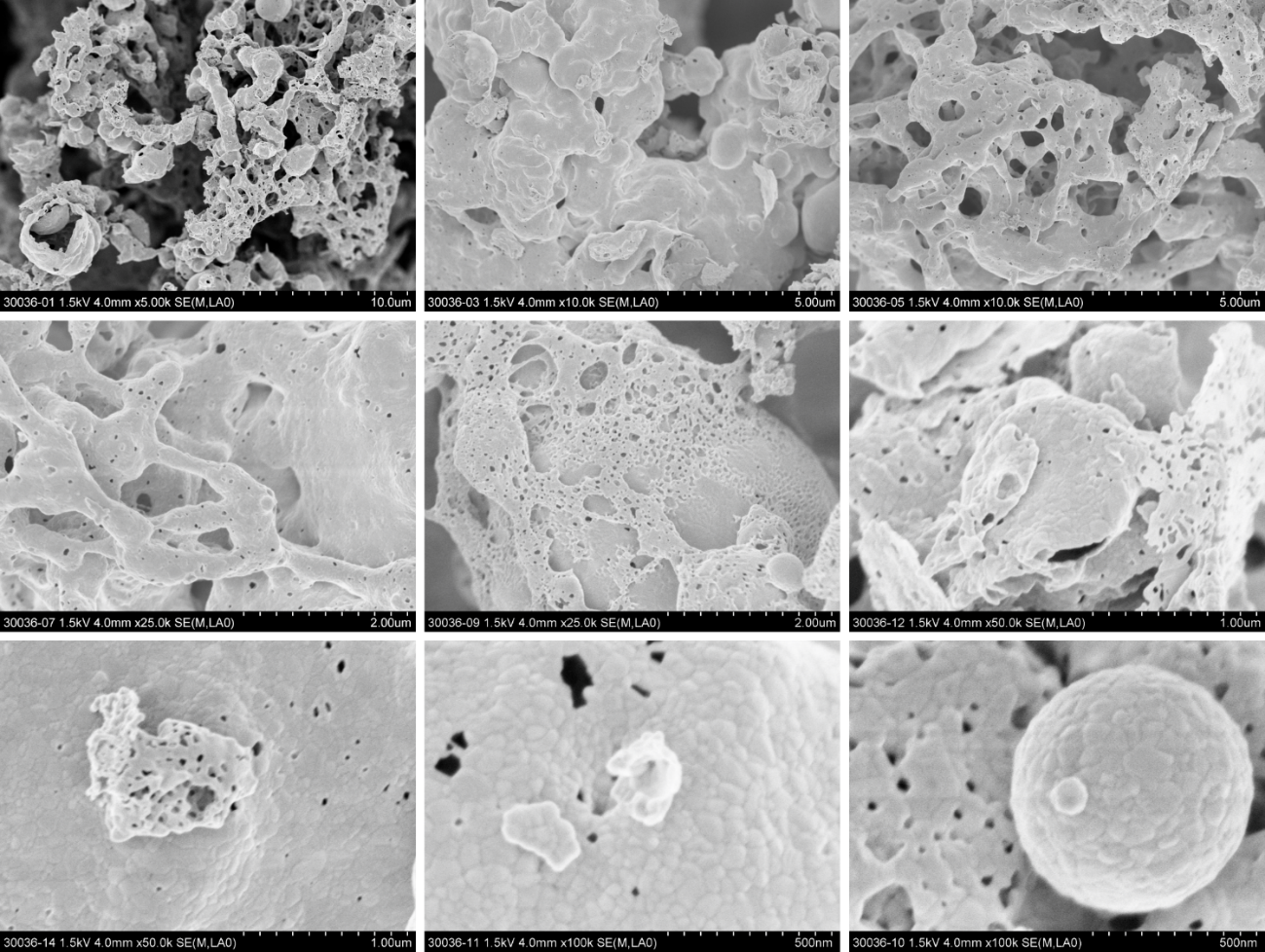


Supplementary Figure S3 Collection of representative SEM images of the fresh calcined Sm_0.96_MnO_3_ catalyst. The catalyst shows various macroscopic structures, a broad range of undefined pores and nanocrystals sticking together and forming particles.


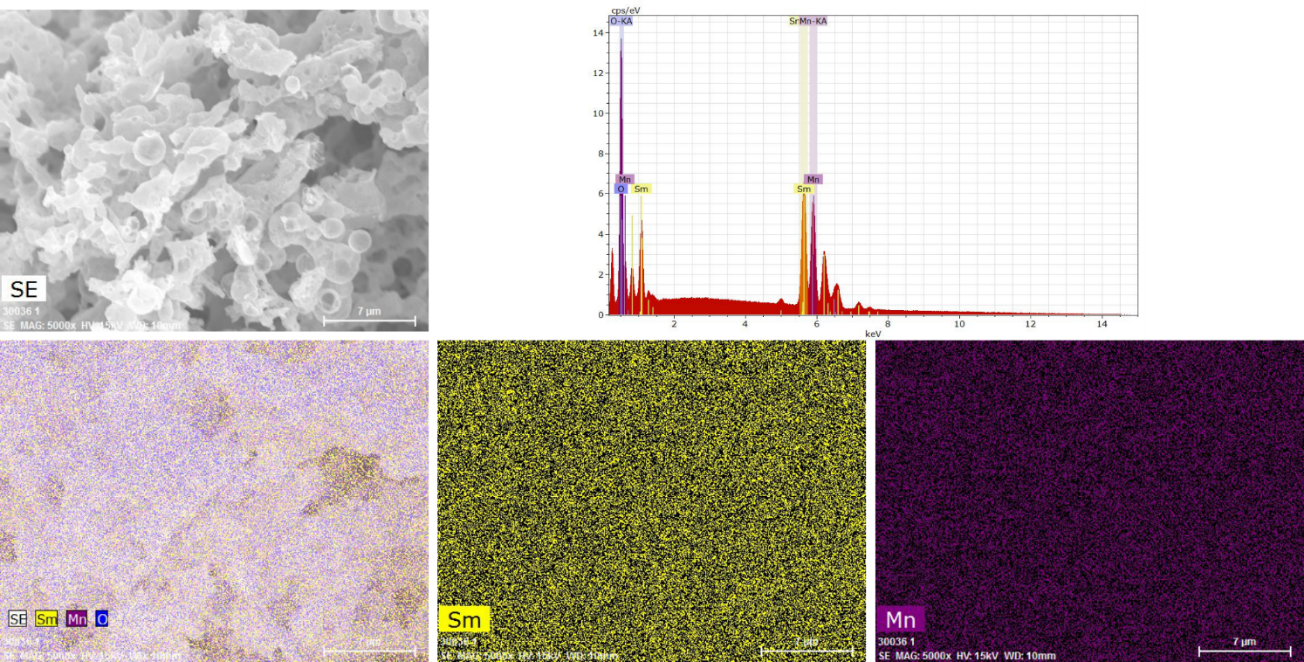


Supplementary Figure S4 Representative map of Sm_0.96_MnO_3_ (top left) and the EDX spectrum (top right). Accumulated elemental distribution of Sm (yellow), Mn (purple) and O (blue) (bottom left) and therefrom extracted elemental distribution of Sm (bottom middle) and Mn (bottom, right).


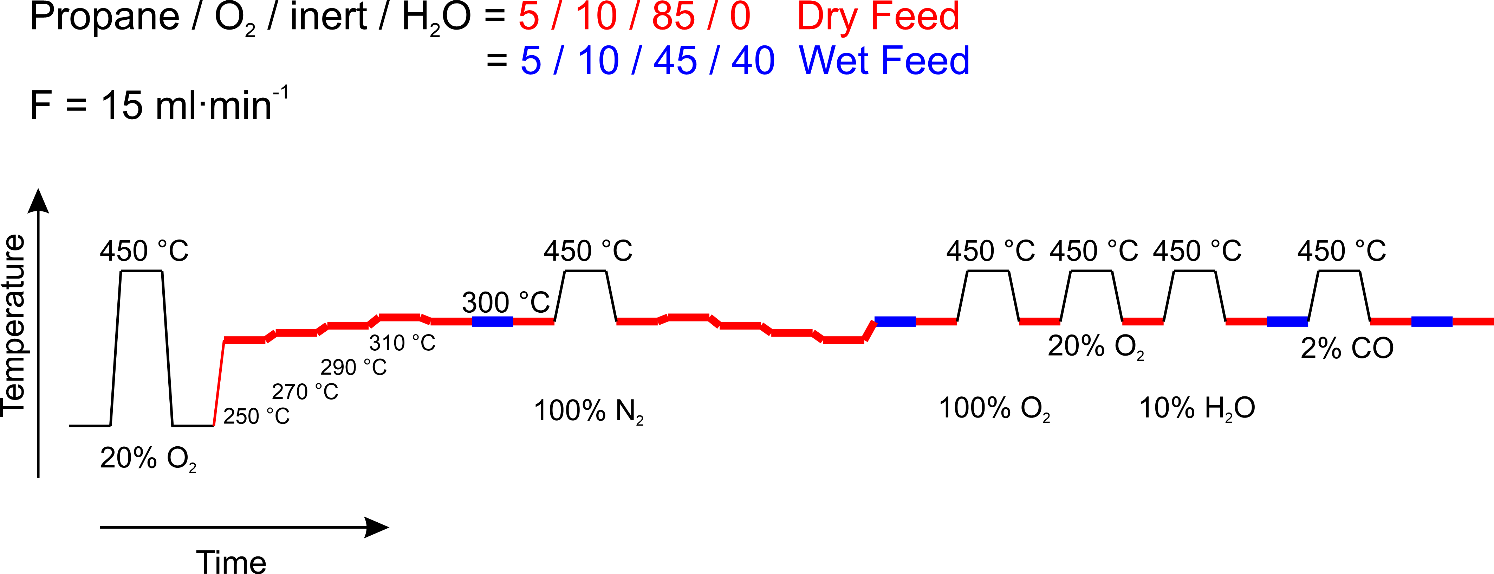


Supplementary Figure S5 Workflow of the reaction conditions applied in the laboratory reactor. Pretreatment steps in different atmospheres at 450 °C are shown in black, propane oxidation in dry feed at a reaction temperature range of 250 °C to 310 °C to is shown in red, and propane oxidation in wet feed at a reaction temperature of 300 °C is shown in blue.





Supplementary Figure S6 Normalized TEY intensities of Sm M_5,4_-edges of Sm_0.96_MnO_3_ collected at different conditions given as C_3_H_8_/O_2_/He/H_2_O (initial – black, 10% O_2_/He – green, wet feed – blue, CO/He – grey, dry feed – red).


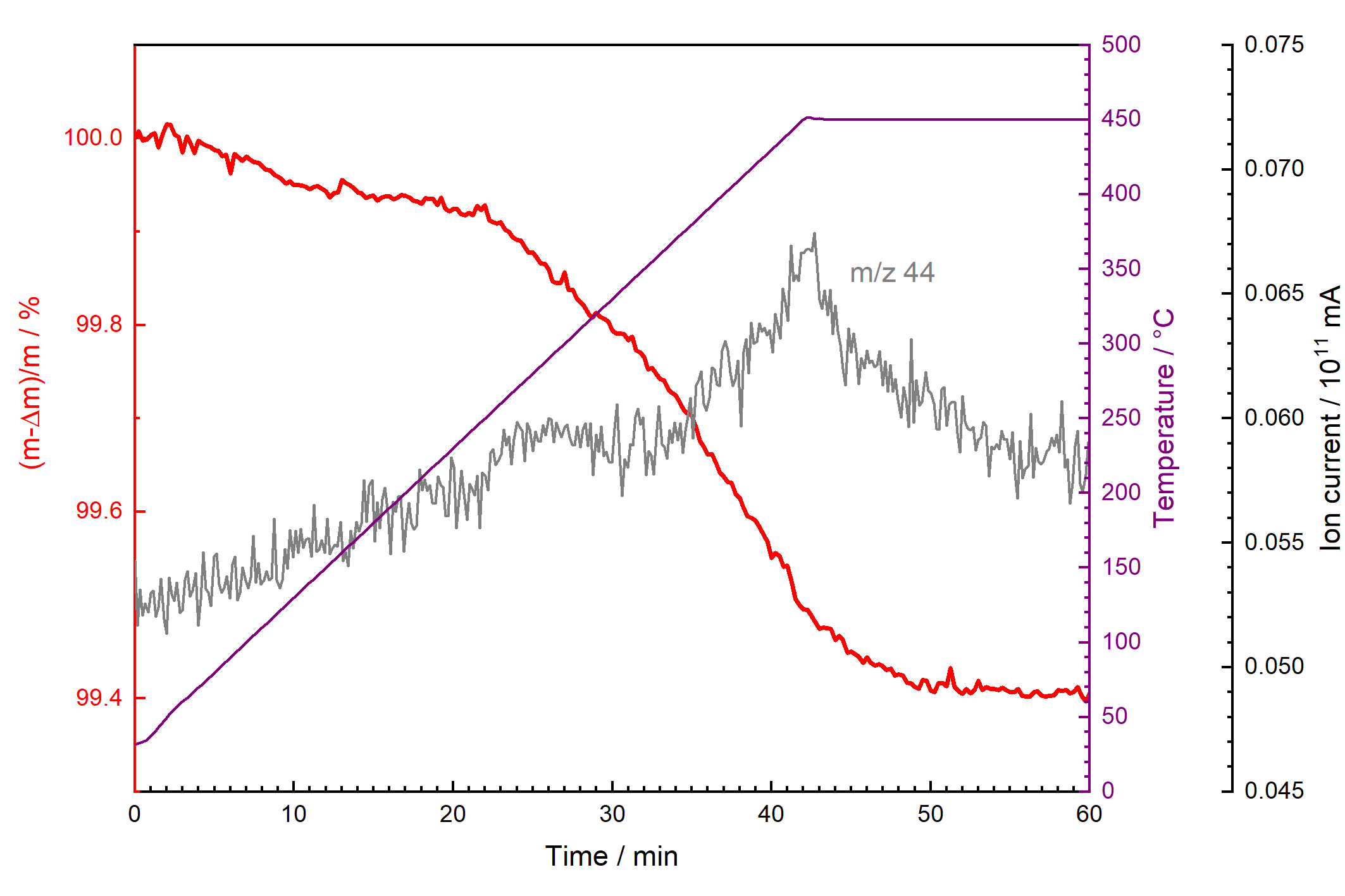


Supplementary Figure S7 Relative mass loss (red), the temperature (purple) and the corresponding ion current of m/z = 44 as an indication for CO_2_ formation. The Atmosphere consisted of 2% CO balanced by Ar. Before measurement, the sample was heated to 450 °C in 21% O_2_ balanced by Ar.


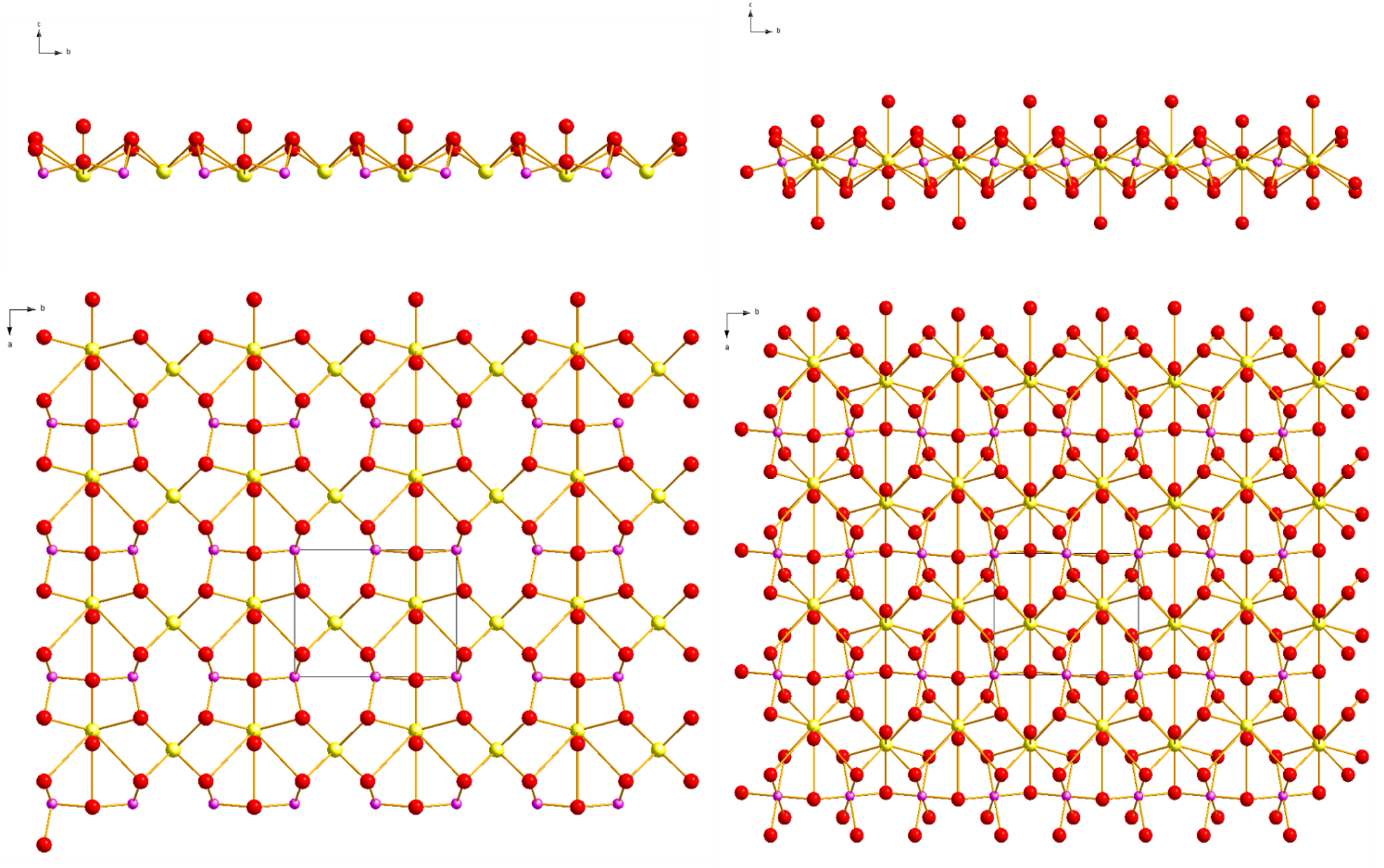


Supplementary Figure S8 The 001 surface of SmMnO_3_ as cross-section at the top and as the top view at the bottom. Mn is represented in purple, Sm in yellow and O in red. On the left: only the outermost surface oxygen atoms are depicted. On the right: all oxygen atoms linked to the outermost surface metal atoms (Mn and Sm) are depicted. Removal of all depicted oxygen atoms would yield in mass loss of 0.2% and 0.6% for left and right representation, respectively.

## Supplementary Tables

Supplementary Table S1 Apparent activation energies E_a_ in the temperature range 250 °C < T < 310 °C measured initially and after treatment in pure N_2_ for different residence times in dry feed.

| Mass / mg | Flow / ml∙min^-1^ | C_3_H_8_/O_2_/N_2_ | W/F / g∙s∙ml^-1^ | E_a_ / kJ∙mol^-1^ | |
| --- | --- | --- | --- | --- | --- |
|  |  |  |  | initial | after N_2_ at 450 °C |
| 52.4 | 15 | 5/10/85 | 0.22 | 67 | 80 |
| 96 | 15 | 5/10/85 | 0.38 | 60.6 | 75.2 |
| 142.4 | 15 | 5/10/85 | 0.57 | 55.3 | 72.5 |

Supplementary Table S2 List of averaged distances between oxygen atoms O1, O2 and Mn, Sm and both together (M) in an octahedral coordination sphere and estimated standard deviations.

|  | Average distance / Å^a^ |
| --- | --- |
| O1-Mn | 1.947 ± 0.000 |
| O1-Sm | 2.872 ± 0.612 |
| O1-M^b^ | 2.564 ± 0.673 |
|  |  |
| O2-Mn | 2.072 ± 0.227 |
| O2-Sm | 2.787 ± 0.681 |
| O2-M^b^ | 2.549 ± 0.588 |
| ^a^ Variance is given as the estimated standard deviation of considered distances from Figure 7  ^b^ M represents both Sm and Mn | |
